# Immunoinformatics design of multivalent epitope vaccine against monkeypox virus and its variants using membrane-bound, enveloped, and extracellular proteins as targets

**DOI:** 10.3389/fimmu.2023.1091941

**Published:** 2023-01-26

**Authors:** Muhammad Waqas, Shahkaar Aziz, Pietro Liò, Yumna Khan, Amjad Ali, Aqib Iqbal, Faizullah Khan, Fahad Nasser Almajhdi

**Affiliations:** ^1^ Department of Biotechnology and Genetic Engineering, Hazara University, Mansehra, Pakistan; ^2^ Natural and Medical Sciences Research Center, University of Nizwa, Birkat-ul-Mouz, Nizwa, Oman; ^3^ Institute of Biotechnology and Genetic Engineering, The University of Agriculture, Peshawar, Pakistan; ^4^ Department of Computer Science and Technology, University of Cambridge, Cambridge, United Kingdom; ^5^ Department of Biotechnology, Abdul Wali Khan University Mardan, Mardan, Pakistan; ^6^ Department of Pharmacy, Abdul Wali Khan University Mardan, Mardan, Pakistan; ^7^ Department of Botany and Microbiology, College of Science, King Saud University, Riyadh, Saudi Arabia

**Keywords:** monkeypox, monkeypox virus, multi-epitope vaccine, multivalent epitope vaccine, reverse vaccinology, immunoinformatics, T-and B-cell

## Abstract

**Introduction:**

The current monkeypox (MPX) outbreak, caused by the monkeypox virus (MPXV), has turned into a global concern, with over 59,000 infection cases and 23 deaths worldwide.

**Objectives:**

Herein, we aimed to exploit robust immunoinformatics approach, targeting membrane-bound, enveloped, and extracellular proteins of MPXV to formulate a chimeric antigen. Such a strategy could similarly be applied for identifying immunodominant epitopes and designing multi-epitope vaccine ensembles in other pathogens responsible for chronic pathologies that are difficult to intervene against.

**Methods:**

A reverse vaccinology pipeline was used to select 11 potential vaccine candidates, which were screened and mapped to predict immunodominant B-cell and T-cell epitopes. The finalized epitopes were merged with the aid of suitable linkers, an adjuvant (Resuscitation-promoting factor), a PADRE sequence (13 aa), and an HIV TAT sequence (11 aa) to formulate a multivalent epitope vaccine. Bioinformatics tools were employed to carry out codon adaptation and computational cloning. The tertiary structure of the chimeric vaccine construct was modeled via I-TASSER, and its interaction with Toll-like receptor 4 (TLR4) was evaluated using molecular docking and molecular dynamics simulation. C-ImmSim server was implemented to examine the immune response against the designed multi-epitope antigen.

**Results and discussion:**

The designed chimeric vaccine construct included 21 immunodominant epitopes (six B-cell, eight cytotoxic T lymphocyte, and seven helper T-lymphocyte) and is predicted non-allergen, antigenic, soluble, with suitable physicochemical features, that can promote cross-protection among the MPXV strains. The selected epitopes indicated a wide global population coverage (93.62%). Most finalized epitopes have 70%–100% sequence similarity with the experimentally validated immune epitopes of the vaccinia virus, which can be helpful in the speedy progression of vaccine design. Lastly, molecular docking and molecular dynamics simulation computed stable and energetically favourable interaction between the putative antigen and TLR4.

**Conclusion:**

Our results show that the multi-epitope vaccine might elicit cellular and humoral immune responses and could be a potential vaccine candidate against the MPXV infection. Further experimental testing of the proposed vaccine is warranted to validate its safety and efficacy profile.

## Introduction

The present monkeypox virus (MPXV) outbreak has resulted in 59, 147 confirmed cases and 23 deaths, spanning 103 countries, as of 14 September 2022, and most of these cases have been detected in countries that do not have previous MPXV transmission instances (1). This distingue the current MPXV outbreak from the previous ones, which were mainly localized (2–4) and had limited person-to-person transmission, causing fewer infections (5–7). World Health Organization (WHO) declared the 2022 MPXV outbreak a worldwide health emergency of international concern on 23 July 2022 (8). MPXV is the etiological agent of monkeypox disease (MPX, a re-emerging zoonotic disease) and falls under the *orthopoxvirus* genus of the family *Poxviridae* (9). This genus also comprises three other human pathogens, such as vaccinia, cowpox, and variola (causative agent of smallpox) (10). MPXV is a membrane-enveloped double-stranded DNA virus containing a ~19.7 kb long genome that encodes 190 open reading frames (ORFs) (11). The virus is comprised of two distinct clades: the central African (Congo-basin) MPXV, with an associated case mortality rate of over 10%, and the west African MPXV, which is reported to be less lethal (fatality rate 3.6%) (12). The underlying causes of the current MPXV outbreak are unclear; nevertheless, phylogenetic analysis of genomic sequences reported on GISAID from at least 15 countries put these isolates in the west African clade of MPXV (13). This is remarkable considering the clade’s low outbreak-causing potential, as reported previously (14–16).

One of the therapeutic interventions to prevent and control MPX is using the vaccinia-virus (VACV) based vaccines, developed initially against smallpox. There are three main classes of VACV-based vaccines. First-generation vaccines include live VACV, such as Dryvax, which has been used to eradicate smallpox in the previous century (17). These vaccines are kept in store by the WHO and numerous other nations; however, their use against MPXV is not advised due to safety concerns (13). Second-generation vaccines, including ACAM2000, are also live VACVs with a better safety profile than first-generation vaccines. Under extended access investigational new drug application (13), the ACAM2000 vaccine is presently available in the US for use against MPX. Since this vaccine is replication-competent (18), there is a likelihood of severe side effects (e.g., progressive vaccinia and eczema vaccinatum) and myopericarditis (estimated incidence of 5.7 per 1,000 primary vaccinees based on clinical trial data). Further, person-to-person transmission of VACV can happen (18, 19). To contain the present MPX outbreak, WHO and the US Centers for Disease Control and Prevention (CDC) currently advise using a third-generation vaccine, Bavarian Nordic’s modified vaccinia virus Ankara (MVA-BN), for high-risk populations. The efficacy results of VACV-based vaccines, however, are published for the MPXV Congo Basin clade (MPXV-CB), and there is a shortage of scientific evidence on the cross-reactive immunity and effectiveness of such vaccines against the MPXV-WA clade of viruses (which seems most relevant to the sequences seen in the present outbreak) (13).

Multi-epitope vaccination offers an emerging solution for preventing and controlling viral infection due to its multiple advantages over the experimental-based approach, such as being timesaving, cost-effective, and convenient (20, 21). Compared to the monovalent vaccine, the multi-epitope vaccine benefits from concurrently activating innate, cellular, and humoral immune responses (22). Thus, designing a multi-epitope vaccine with broad-spectrum application potential for MPXV and its emerging variants could offer a promising therapeutic option to tackle the evolving infection. In the present study, we aimed to computationally design and evaluate a broad-spectrum multi-epitope vaccine that can elicit humoral and adaptive immune responses against MPXV infection targeting the membrane-bound, enveloped, and extracellular proteins of the virus.

## Methods

### Extraction of monkeypox proteins and preliminary analysis

**Figure 1:**
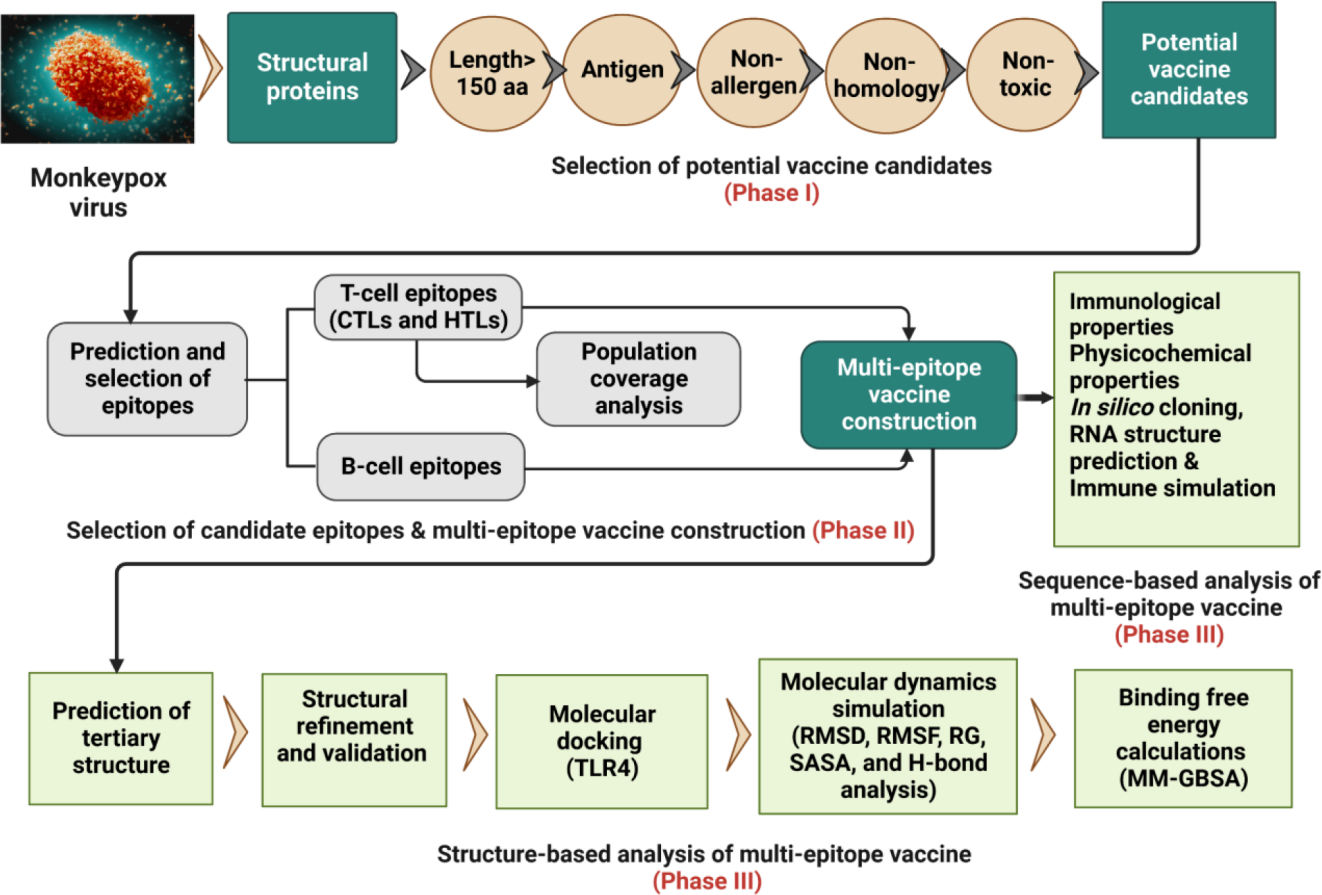
Schematic representation of the research pipeline implemented in this study.to design a multi-epitope vaccine construct for the MPXV.

The whole proteome of the reference MPXV Zaire-96-I-16 (RefSeq assembly accession: GCF_000857045.1) was retrieved from the NCBI genome database (https://www.ncbi.nlm.nih.gov/genome/5259) in FASTA format. Further, protein sequences (FASTA format) of recently identified six MPXV isolates, including USA (Accession ON674051.1), UK (Accession MT903345.1), Japan (Accession LC722946.1), Germany (Accession OP263629.1), Finland (Accession ON959143.1), and Canada (Accession ON736420.2) were downloaded from the NCBI GenBank (https://www.ncbi.nlm.nih.gov/genbank/). The overall workflow to design a multi-epitope vaccine for MPXV is shown in **Figure 1**.

### Selection of potential vaccine candidates

UniProt database (https://www.uniprot.org/uniprotkb?query=Monkeypox%20virus) was checked to identify and select only membrane-bound, enveloped, or extracellular proteins of MPXV. Those proteins with at least 150 amino-acids residues were selected for downstream analysis. The collected proteins were checked for antigenicity and non-allergen character using the VexiJen v2.0 (23) (threshold value 0.5) and AllerTOP v.2.0 (24), respectively. Besides, the ToxinPred server (25) was employed to select proteins predicted as non-toxic. Finally, the proteins were passed through a similarity check with Homo sapiens proteome (NCBI Taxid: 9606) *via* BLASTp (https://blast.ncbi.nlm.nih.gov/Blast.cgi?PAGE=Proteins) to select non-human homologue having a sequence identity ≤ 35%.

### Prediction of T-cell epitope

The finalized protein sequences were submitted to the IEDB (NetMHCpan BA 4.1 method) (http://tools.iedb.org/mhci/) and NetMHCIIpan-4.0 server (26) to predict cytotoxic T lymphocyte [(CTL) also called CD8+ T-cell or major histocompatibility complex (MHC)-II binding epitopes)] and helper T lymphocyte [(HTL) also called CD4+ T-cell or MHC-I binding epitopes)] epitopes, respectively. The reference sets provided by these servers with the highest population coverage and human leukocyte antigen (HLA) allele frequencies were used to predict epitopes. In case of CTL epitopes, the dataset comprised 77 alleles. HTL epitopes were tested for binding affinities with commonly occurring HLA class II alleles from the provided dataset: HLA-DRB1-0101, HLA-DRB1-0301, HLA-DRB1-0401, HLA-DRB1-0701, HLA-DRB1-0801, HLA-DRB1 0901, HLA-DRB1-1001, HLA-DRB1-1101, HLA-DRB1-1201, HLA-DRB1-1301, HLA-DRB1-1401, HLA-DRB1-1501, and HLA-DRB1-1601 (27). The selected HLA class I alleles automatically limit the available peptide length. Epitopes showing the IC_50_ value<50nM [high-affinity binders (28)] with at least three reference set alleles were retained. On the other hand, 15-mer epitopes predicted as strong binding peptides with a minimum of three HLA class II reference sets were chosen for further analysis.

### Prediction of sequential b-cell epitopes

The amino acid sequence of selected proteins of MPXV was used to predict the linear B-cell epitopes using the ABCpred server (primary server) (29). Applying the server threshold of 0.71, epitopes with a 20-mer length were selected for the next stage. The selected protein sequences were also submitted to Bepipred-2.0 (30) and LBtope (selected model “LBtope_Fixed”) (31) servers, using the default threshold values, to check whether we obtain the same epitopes predicted by the primary server.

Only B-cell epitope sequences confirmed by these three servers were deemed potential epitopes and were subjected to additional analysis.

### Epitopes antigenicity, allergenicity, immunogenicity, and human homology

Using the VexiJen v2.0 server (23), the ability of previously chosen epitopes to act as an antigen was tested. For the “virus” model, epitopes with an antigenicity score of 0.5 or above were selected for the next evaluation. To evaluate the allergenicity of epitopes, the AllerTOP v.2.0 server (24) was employed. The immunogenicity of CTL epitopes was also assessed through the IEDB’s Class I Immunogenicity tool (32), applying the default parameters. To assess the epitopes’ similarity with human proteins and thus minimize the chance of autoimmunity, a BLASTp was performed. The amino acid sequence of the individual epitope was submitted against the target organism selected as “Homo sapiens (NCBI Taxid:9606)”. Epitopes that showed<70% identity with human proteins (query coverage above 70%) were considered appropriate for subsequent analysis.

### Selecting epitopes that overlapped with experimentally validated epitopes

Here, we used the shortlisted B-cell and T-cell (CTL and HTL) epitopes to search for overlapping epitopes at ≥70% identity from the IEDB epitope database (33, 34). The overlapped epitopes (resulting from other pathogens) could accelerate the vaccine candidates’ immunogenicity assessment and observation of potential outcomes that result from mutations and epitope escape while the virus spreads through the human population (35).

### IFN-γ and interleukins secretion potential of epitopes

IFN-γ inducing potential of the finalized HTL epitopes was predicted through the “predict” module of the IFNepitope server (36). We selected “Motif and SVM hybrid” and “IFN-γ versus non–IFN-γ” as a prediction approach and model, respectively. Epitopes with a positive prediction score were considered IFN-γ inducing epitopes. Moreover, the interleukin-4 (IL-4) and interleukin-10 (IL-10) induction potential of the chosen HTL epitopes were evaluated *via* IL4pred (37) (threshold value 0.2) and IL10pred server (38) (threshold value 0.2), respectively.

### Multiple sequence alignment

To check the selected epitopes conservancy across various MPXV variants, the FASTA sequence of the final candidates B-cell and T-cell epitopes were aligned with their source protein sequence from the collected six MPXV isolates. Clustal Omega (39) program was used with default settings to carry out multiple sequence alignment.

### Construction of multi-epitope vaccine ensemble

Suitable linkers and an adjuvant was used to fuse the selected epitopes in a rationally immunogenic manner. The explicit linkers were used to connect different vaccine components, including EAAAK, GGGS, AAY, GPGPG, and AAY. The N-terminal region of the construct begins with HIV-TAT (TGALLAAGAAA) peptide that facilitates cell penetration. Next, we choose an adjuvant, Resuscitation-promoting factor, RpfE (UniProt Entry: O53177), to improve the immunogenicity of the vaccine ensemble. Then, the Pan DR epitope (PADRE – AKFVAAWTLKAAA) was added to play an HTL stimulus role. Subsequently, CTL epitopes were fused, followed by HTL and B-cell. Finally, the 6xHis tag was appended at the C-terminal of the construct to assist the purification assays.

### Evaluation of antigenicity, allergenicity, and toxicity

Antigenicity assessment of multi-epitope vaccine was carried out employing the VexiJen v2.0 (23) (threshold score of 0.5, selected model “virus”) and ANTIGENpro (https://scratch.proteomics.ics.uci.edu/) server. In addition, the AllerTOP v.2.0 (24) and AllergenFP v.1.0 (40) server was used to evaluate the allergenicity of the vaccine construct. The toxicity of the designed vaccine ensemble and its components (epitopes) was assessed with the aid of the ToxinPred server (25).

### Proteasomal processing, relative surface accessibility, and population coverage analysis

Following the multi-epitope vaccine construction, we wanted to confirm its processing inside the cell and that the MHC-1 epitopes would be produced. This was performed by exploiting the NetChop-3.1 server (41) for locating the proteasomal cleavage sites. Besides, the surface accessibility of the amino acids in the constructed vaccine was predicted with NetSurfP-3.0 sever (42). The IEDB population coverage tool (http://tools.iedb.org/population/) was exploited to estimate the coverage of vaccine epitopes in the target population. Herein, we focused on collective population coverage of HLA binding alleles of CTL and HTL epitopes worldwide and across multiple contents.

### Physicochemical properties estimation

Employing the ProtParam server (https://web.expasy.org/protparam/), a set of physicochemical parameters were estimated for the chimeric vaccine and selected epitopes. The solubility of the designed vaccine was also assessed *via* the SOLpro (43) and Protein-Sol (44) servers. Furthermore, transmembrane helices in the constructed vaccine were predicted using the TMHMM v2.0 tool (45). Additionally, the amino acid sequence of the designed vaccine construct was checked for homology with proteins of the human proteome using the BLASTp tool.

### *In silico* immune assay for vaccine efficacy

*In silico* immune simulation was performed, deploying the C-ImmSim server (46) further to characterize the multi-epitope vaccine’s immune response and immunogenicity. The server is an agent-based model that implements a position-specific scoring matrix (PSSM) for epitope prediction and a machine-learning algorithm to forecast immune interactions. Considering the literature (47, 48), a total of 100 simulation steps (a single time-step is equivalent to 8h of daily life) were used, and the injection time-step was set to 1. All other simulation parameters were retained as default.

### Prediction, refinement, and quality assurance of tertiary structure

To predict the multi-epitope vaccine’s three-dimensional (3D) structure, we used the RoseTTAFold server (49). For improvement in the local and global structure quality, the predicted 3D structure was submitted to GlaxyRefine (https://galaxy.seoklab.org/cgi-bin/submit.cgi?type=REFINE) server. This tool applies dynamics simulations to carry out structural perturbations and relaxations. The refined structure was validated using the Molprobity server-generated Ramachandran plot analysis (50) to identify the number of residues in the favoured, allowed, and outlier regions. Moreover, the protein structural validation process was finalized with the utility of the ProSA-web server [28].

### Prediction of discontinuous B-cell epitopes

A protein folding event can bring the distant residues into proximity, forming discontinued B-cell epitopes. As per estimation, over 90% of B-cell epitopes are discontinues one (51). The vaccine’s refined and validated 3D protein structure was input to the ElliPro server (51) (keeping the default settings) to predict the presence of such epitopes.

### Epitopes modeling and docking with MHC receptor

Employing the PEP-FOLD 3.0 server (52), the selected CTL and HTL epitopes were modelled with 2000 simulations and sOPEP sorting scheme. Molecular docking was performed between each epitope and the corresponding HLA allele (picked on the basis of best affinity) using the protein–protein (P–P) docking protocol of MOE2020 software (53). Crystallographic structures of HLA class I molecules, including HLA-B*15:01 (PDB ID: 3C9N), HLA-A*11:01 (PDB ID: 5WJN), HLA–B*35:01 (PDB ID: 4PR5), HLA–A*30:01 (PDB ID: 6J1W), HLA-B*15:02 (PDB ID: 6UZM), HLA-B*58:01 (PDB ID: 5VWH), HLA-A*02:06 (PDB ID: 3OXR) and HLA class II molecules, such as HLA-DRB1*01:01 (PDB ID: 1AQD), HLA-DRB1*04:01 (PDB ID: 5NI9), and HLA-DRB1*11:01 (PDB ID: 6CPN) were collected from RCSB Protein Data Bank (https://www.rcsb.org/). Prior to docking, extracted PDB structures were energy minimized with the Quickprep module of MOE2020 software (53), and attached ligand molecules were removed. The binding site of the attached peptide with the crystal structure of the HLA molecule was selected as a target site for docking the epitopes. Interface contact analysis of epitope–receptor was done through the P–P interaction panel of MOE2020 (53).

### Molecular docking of modelled vaccine with TLR4

Molecular docking was carried out to test the binding affinity of the designed vaccine for the Toll-like receptor 4 (TLR4). For this analysis, TLR4 structure was extracted from the RCSB Protein Data Bank (PDB ID: 3FXI). Next, the crystal structure was edited to remove the attached oligosaccharides and ligands, and was energy minimized. The rigid body refinement algorithm of MOE2020 was used to retain the final 30 docked poses of the docked complex. Utilizing the PDBsum server [24] and Blender software (54), the vaccine construct–receptor interface contacts and 3D structural illustrations were generated, respectively. The best-docked confirmation of vaccine construct–TLR4 complex based on a high docking score (DS) was subjected to molecular dynamics (MD) simulation analysis.

### Molecular dynamics simulations of vaccine–TLR4 complex

MD simulations of the best-docked pose of the vaccine and TLR4 complex were carried out using the implicit solvent approach of AMBER22 (55). Applying the residue-specific ff19SB [7] forcefield, the coordinates and topology file of the protein were prepared. To maintain the systems as neutral, ~0.1 M concentration of Na^+^ and Cl^-^, also called monovalent optimal point charge (OPC) ions, were added with a grid size of 1Å (56). With the utility of the LEap component of AMBER22, missing hydrogen (H) atoms were added to the protein residues. Each system was solvated in an OPC water model inside a truncated octahedral box with a 10Å of buffer distance. In order to accelerate paralleled scaling during the calculations of long-range electrostatics, the Particle Mesh Ewald Molecular Dynamics (PMEMD) (57) engine of AMBER22 with a GPU version was implemented. A two-step energy minimization of each system was done for MD. First, energy minimization using the steepest descent algorithm was performed for 2000 steps. Next, the systems’ energy was minimized with the conjugate gradients algorithm for 10000 steps (58). Using the Langevin thermostat (59), each system was gradually heated from 0.1K to 300K during the course of a 400ps timescale in a microcanonical ensemble (NVE) while applying weak restraints on the P–P complex. The same thermostat was also used with a collision frequency of 2.0ps^-1^ to adapt the protein kinetic energy of harmonic oscillators for dynamic propagation. The heating approach described earlier was followed for the adjustment of density in 400ps time. An NPT ensemble was employed to equilibrate the systems at 300K during the 2000ps time. To regulate the temperature during the equilibration stage, the Langevin dynamics was exploited. Besides, a constant pressure was applied (with a relaxation time of 2ps) at this stage. Long-range electrostatics were computed with a distance cut-off of 8Å. All dynamics simulations were done with H atoms experiencing the SHAKE and a 2fs time-step (60). The protocol followed for the equilibration stage was used to finally perform 100ns production MD run for the modelled vaccine and TLR4 complex. The trajectory generated every 10ps was gathered for further analysis.

### Evaluation of post-dynamics

The trajectory produced from the simulations was subjected to stability analysis of the protein complex concerning the root mean square deviation (RMSD) and root mean square fluctuation (RMSF). The CPPTRAJ package (61) of the AMBER22 was employed for this analysis, taking only the cartesian coordinates of C-alpha atoms. The radius of gyration (Rg) was also computed to evaluate the structural compactness of the protein complex by applying the equation described in (61). In addition, the solvent-accessible surface area (SASA) was computed to examine the surface accessibility of protein to the solvent molecules. We also computed constructed vaccine–TLR4 interface hydrogen bonds (H-bonds) because they influence the stability of the protein. We set acceptor–donor distance and angle cut-offs of 3.5Å and 120°, respectively, for the H-bond analysis.

### Binding free calculation of the putative vaccine–TLR4 complex

To calculate the binding free energy of the modelled vaccine–TLR4 complex, the MM/GBSA (Molecular Mechanics/Generalized Born Surface Area) approach was used (62). By applying equation 1, the free energy of each complex was calculated (63).


(Equation 1) ΔGbind = ΔGR+L – (ΔGR + ΔGL) 

ΔGR+L is the protein-ligand complex energy, while ΔGR and ΔGL is the apoprotein and ligand energy (vaccine construct), respectively. Each ΔG term (free energy) given in equation 1 was computed by applying the equations described in (64).

### Computational cloning and prediction of RNA secondary structure for the designed vaccine

To obtain the designed vaccine’s cDNA (nucleotide sequence), its amino acid sequence was input into the EMBOSS Backtranseq (https://www.ebi.ac.uk/Tools/st/emboss%20backtranseq/) tool. Next, the obtained codon was adapted for usage by *E. coli* (strain K-12*)* cellular machinery using the Java Codon Adaptation Tool (JCat) server (65). To ensure proper insertion into the plasmid, restriction sites of XhoI and NheI were appended, flanking the adapted codon sequence of the construct. Subsequently, the adapted sequence was cloned in the pET28a (+) expression vector utilizing the SnapGene software (https://www.snapgene.com/). Moreover, the adapted cDNA sequence of the designed vaccine was submitted to the RNAfold program (66) to predict the mRNA secondary structure with the least free energy.

## Results

### Selection of target proteins

UniProt search provided 37 integral membrane proteins, three envelope proteins, and four proteins located in the extracellular space for MPXV. Out of it, 11 proteins, including eight integral membrane proteins, two extracellular (secretory) proteins, and a single envelope protein of the virus, were selected based on several desirable properties (**Table 1**).

**Table 1:** Final candidate proteins of MPXV selected for epitope mapping.

NCBI ID	Protein ID	Length (>150 aa)	VexiJen score (≥0.5) ^A^	Allergenicity	BLASTp (Accession No.) ^C^	Cellular location	Reference for cell location
NP_536572.1	A35R	181	0.53	Non-A ^B^	Non-significant	Integral component of membrane	UniProt (Q80KX2) **^D^**
NP_536580.1	A43R	197	0.54	Non-A	Non-significant	Integral component of membrane	Uniprot (F1DJ14)
NP_536597.1	B9R	267	0.54	Non-A	Non-significant	Secreted IFN- γ binding protein	UniProt (Q5IXK7)
NP_536604.1	B16R	352	0.54	Non-A	Non-significant	IFN-alpha-beta-receptor-like protein secreted glycoprotein/IFN-alpha/beta receptor glycoprotein	UniProt (Q5IXK2)
NP_536457.1	C4L	424	0.54	Non-A	31% (NP_001307201.1)	Viral envelope protein	UniProt (Q5IXZ5)
NP_536464.1	C11L	343	0.55	Non-A	Non-significant	Integral component of membrane	Uniprot (Q3I8X9)
NP_536444.1	D14L	216	0.62	Non-A	31% (BAG52091.1)	Integral component of membrane	UniProt (Q98VL5)
NP_536532.1	E8L	304	0.52	Non-A	35% (NP_001730.1)	IMV surface membrane 32 kDa protein	UniProt (Q8V4Y0)
NP_536537.1	E13L	551	0.52	Non-A	Non-significant	Membrane; Peripheral membrane protein, viral capsid	UniProt (Q5IXR5)
NP_536506.1	G10R	340	0.51	Non-A	Non-significant	Integral component of membrane	UniProt (Q8V503)
NP_536609.1	B21R	1879	0.51	Non-A	Non-significant	Integral component of membrane	UniProt (V9NT24)

^A^antigenicity score provided by the VexiJen v2.0 server. The obtained score of ≥ 0.5 indicates the protein is a probable antigen^B^non-allergen ^C^accession number of the protein that showed the highest similarity with the MPXV protein sequence in the BLASTp output (Percent identity is reported for that protein)**^D^** UniProt entry (RefSeq assembly accession: GCF_000857045.1: RefSeq accession: NC_003310.1M; ID: 5259).

### Selection of final candidate cytotoxic T- lymphocyte epitopes

IEDB server predicted 78 promiscuous CTL epitopes that presented high affinity (IC_50_<50 nM) for ≥ 3 HLA class I alleles. We further filtered 13 epitopes based on positive immunogenicity score, non-allergen, non-toxic, antigen property, and non-homology. To select the final candidates, the epitopes were checked for sequence similarity with the experimentally determined epitopes of other organisms submitted to the IEDB. We found a high similarity of the chosen eight CTL epitopes sequences with the experimentally determined epitopes of the vaccinia virus, except for IAYRNDTSF, which resembles *Brucella abortus* 544 epitopes. It is pertinent to mention that KMRDTLPAK and KTFAIIAIV show 100% sequence resemblance with experimentally validated epitopes of vaccinia virus. The predicted antigenicity score of the final candidates ranged from 0.59–1.39. Physicochemical properties of epitopes indicated that peptides have molecular weights ranging from 975.24Da to 1108.31Da; half are predicted alkaline and as many with slightly acidic pI. The antigen source, high-affinity HLA I alleles, and various predicted properties of the eight CTL candidate epitopes are provided in the **Table 2**.

**Table 2:** Potential promiscuous CTL epitopes identified for the most frequent HLA class I alleles.

Antigen	CTL epitope	Position ^A^	IEDB Predictions MHC-I allele (IC50<50nM)	IEDB Immuno-genicity score	VexiJen Score (≥ 0.5) ^B^	BLASTp % Identity (Accession No.) ^C^	MW ^D^	pI ^E^
A35R	LSMITMSAF	46–54	HLA-B*15:25(4.33), HLA-C*03:02(4.77) HLA-B*15:01(7.3), HLA-C*16:01(18.91) HLA-B*58:01(19.9), HLA-B*15:02(20.32) HLA-C*12:02(47.76), HLA-C*03:04(48.04)	0.16	0.75	66.67% (MOL40849.1)	1000.24	5.52
E13L	CINNTIALK	119–127	HLA-A*11:01 (32.15), HLA-A*03:01 (36.77), HLA-A*68:01 (38.2)	0.18	0.94	66.67% (XP_011542496.1)	989.21	8.22
A43R	MSIMPVLTY	1–13	HLA-C*03:02 (4.7), HLA-B*15:25 (5.08), HLA-B*35:01 (9.83), HLA-C*16:01 (10.34), HLA-B*15:02 (13.75), HLA-B*15:01 (14.71), HLA-A*29:02 (16.39), HLA-B*58:01 (16.83), HLA-C*12:02 (35.13)	0.09	0.82	55.56% (EAX07878.1)	1054.33	5.27
B21R	IAYRNDTSF	952–960	HLA-C*16:01 (14.46), HLA-B*15:25 (16.7), HLA-B*35:01 (39.58), HLA-C*03:02 (6.36)	0.001	1.16	66.67% (AAY18488.2)	1086.17	5.84
B16R	KMRDTLPAK	37–45	HLA-A*30:01 (3.47), HLA-A*03:01 (10.45), HLA-A*31:01 (26.96)	0.07	1.39	63.64% (EAW82711.1)	1059.29	9.99
E8L	YVLSTIHIY	61–69	HLA-A*29:02 (6.8), HLA-C*03:02 (14.57), HLA-B*15:25 (17.1), HLA-B*35:01 (18.87), HLA-B*15:02 (28.21)	0.09	0.59	66.67% (EAX02185.1)	1108.31	6.74
E8L	RSANMSAPF	142–150	HLA-B*15:25 (7.31), HLA-C*03:02 (16.81), HLA-C*16:01 (18.61), HLA-B*58:01 (20.56), HLA-A*32:01 (21.43)	0.29	0.95	66.67% (MON86190.1)	980.11	9.75
E8L	KTFAIIAIV	274–282	HLA-A*02:06 (14.3), HLA-A*68:02(34.78), HLA A*30:01(48.78)	0.44	1.02	66.67% (KAI2599878.1)	975.24	8.75

^A^ corresponds to the range (first and last amino acid residue) occupied by the epitope in the source protein ^B^antigenicity score provided by the VexiJen v2.0 server. The obtained score of ≥ 0.5 indicates that the epitope is a probable antigen **^C^** accession number of the protein that showed the highest similarity with epitope sequence in the BLASTp output (Percent identity is reported for that protein). ^D^molecular weight ^E^ theoretical isoelectric point (pI). The chosen epitopes are non-toxic (ToxinPred) and non-allergen (AllerTOP v.2.0).

### Selection of final candidates helper T-lymphocyte epitopes

Considering a set of 14 high-frequency alleles, 81 HTL epitopes were projected as strong binding peptides with at least three HLA class II alleles. We retained only 11 non-allergen, antigenic, non-toxic, and non-homologue epitopes for sequence mapping against the experimentally validated epitopes. Consequently, four of the finalized seven epitopes sequences resemble the vaccinia virus epitopes. At the same time, MNFIPIIYSKAGKIL, SPINIETKKAISDTR, and SLPYKYLQVVKQRER have similarities with the *Brucella abortus* 544, Dengue virus, and Human beta herpes virus 6B epitopes, respectively. The estimated molecular weight for the final candidates varies from 1599.81 to 1907.25Da, and most are predicted to have an alkaline pI. Remarkably, all finalized epitopes are predicted to induce IFN-γ response, and 5/7 HTL epitopes indicated secretion potency of IL-4 and IL-10, as reported in **Table 3**.

**Table 3:** Potential promiscuous HTL epitopes identified for the most frequent HLA class II alleles.

Antigen	HTL epitope	Position ^A^	Interacting MHC class-II allele ^B^	Affinity (nM)	VexiJen Score ^C^	BLASTp % Identity (Accession No.) ^D^	IFN- γ ^E^	IL-4 ^F^	IL-10 ^G^	MW ^H^	pI ^I^
A43R	LIVIIYVFKKIKMNS	183–197	DRB1*0801	92.27	0.60	60.00% (EAX05684.1)	+ve	–ve	+ve	1809.33	10
			DRB1*1101	34.35							
			DRB1*1301	20.62							
			DRB1*1401	98.51							
B16R	FGVYSILTSRGGITE	301–315	DRB1*0101	8.87	0.71	66.67% (BAD92812.1)	+ve	–ve	+ve	1599.81	6
			DRB1*0401	65.14							
			DRB1*1001	18.45							
			DRB1*1101	59.93							
			DRB1*1601	55.86							
C4L	VEVRYIDITNILGGV	99–113	DRB1*0101	35.56	1.43	55.56% (KAI2579105.1)	+ve	+ve	+ve	1660.93	4.37
			DRB1*0401	53.70							
			DRB1*1001	42.15							
			DRB1*1101	227.86							
			DRB1*1601	184.40							
	MNFIPIIYSKAGKIL	239–252	DRB1*0701	5.17	1.13	52.38% (BAB14198.1)	+ve	+ve	+ve	1708.14	9.7
			DRB1*0901	13.38							
			DRB1*1401	48.72							
			DRB1*0101	8.86							
E8L	SPINIETKKAISDTR	6–20	DRB1*0301	741.19	1.06	61.54% (KAI2585319.1)	+ve	+ve	+ve	1672.90	8.31
			DRB1*0801	734.91							
			DRB1*1101	858.56							
			DRB1*1301	195.69							
			DRB1*1401	850.16							
E13L	IRDQYITALNHLVLS	58–72	DRB1*0401	44.23	0.73	61.54% (NP_114089.1)	+ve	+ve	–ve	1756.03	6.74
			DRB1*1001	0.36							
			DRB1*1401	0.29							
			DRB1*1601	0.11							
			DRB1*0701	0.24							
B21R	SLPYKYLQVVKQRER	47-61	DRB1*0101	16.39	1.11	64.29% (AAH04394.1)	+ve	+ve	–ve	1907.25	9.99
			DRB1*0401	56.24							
			DRB1*0801	67.90							
			DRB1*1001	13.06							
			DRB1*1101	36.25							
			DRB1*1601	116.78							

^A^corresponds to the range (first and last amino acid residue) occupied by the epitope in the source protein ^B^ Strong binding alleles (ranked as top 1%) ^C^ antigenicity score provided by the VexiJen v2.0 server. The obtained score of ≥ 0.5 indicates that the epitope is a probable antigen ^D^accession number of the protein that showed highest similarity with epitope sequence in the BLASTp output (Percent identity is reported for that protein).^E^IFN-gamma ^F^interlukin-2 ^G^interleukin-10 ^H^molecular weight ^I^theoretical isoelectric point (pI) The chosen epitopes are non-toxic (ToxinPred) and non-allergen (AllerTOP v.2.0). Interacting alleles are top 1% binder (strong binder) according to the NetMHCpan II server output.

### Selection of final candidates linear B-cell epitopes

The analysis generated a total of 80 sequences confirmed using multiple tools. Twenty-seven epitopes were shortlisted and predicted to be an antigen, non-allergen, non-toxic, and dissimilar to the human proteins. Finally, six linear B-cell epitopes sequences were found to have a high-degree similarity with experimentally validated epitopes of the vaccinia virus. They were deemed final components for vaccine conception and enlisted in **Table 4**. The molecular weight of all final candidates is >1900 Da, and predicted pI classifies them as alkaline. Sequence homology of B-cell and T-cell epitopes with experimentally validated epitopes of other pathogens (homologous epitopes at ≥ 70% identity) is provided in **Table 5**.

**Table 4:** The amino acid sequence of final candidates B-cell epitopes for vaccine construction.

Antigen	B-Cell epitope	Position ^A^	ABCpred Score	VexiJen ^B^ (≥ 0.5)	BLASTp % Identity (Accession No.) ^C^	MW ^D^	pI ^E^
A35R	***STHRKVASSTTQYDHKESCN***	82–101	0.76	0.61	50.00% (EAX07215.1)	2279.43	7.95
G10R	***YVHIGPLTKDKEDKVKKRYP***	42–61	0.76	0.90	43.48% (EAW53731.1)	2414.83	9.6
	***VNTGPGGLSALLRQSYNGTA***	66–85	0.75	0.82	63.16% (AEX29256.1)	1976.18	8.72
E8	VHWN ***KKKYSSYEEAKKHDDG***	94–113	0.83	0.59	55.00% (BAG62355.1)	2449.66	8.29
	***YFMKWLSDLREACFSYYQKY***	250–269	0.79	0.57	45.83% (NP_987099.1)	2642.04	8.12
C4L	**VYW *YLGVNNLPYNWKNFYPS***	164–183	0.83	0.94	50.00% (AAD14242.1)	2537.86	8.35

^A^corresponds to the range (first and last amino acid residue) occupied by the epitope in the source protein ^B^antigenicity score provided by the VexiJen v2.0 server. The obtained score of ≥ 0.5 indicates the epitope is a probable antigen ^C^accession number of the protein that showed highest similarity with epitope sequence in the BLASTp output (Percent identity is reported for that protein). ^D^molecular weight ^E^theoretical isoelectric point. Twenty amino acid long sequence is that of ABCpred predicted epitope: italicized and bold letters is Bepipred predicted epitope sequence; Underline sequence represents LBtope predicted B-cell epitope sequence. The chosen epitopes are non-allergen (AllerTOP v.2.0) and non-toxic (ToxinPred).

**Table 5:** Homology of selected B-cell and T-cell peptides with experimentally validated epitopes of other pathogens (homologous epitopes at ≥ 70% identity).

Epitope Type	Sequence	Homologous Epitope	Homologous Epitope IEDB ID	Organism
CTL	LSMITMSAF	**LSMITMSAF** LIVRLN	85862	Vaccinia virus
	CINNTIALK	N **CINNTIAL**	43374	Vaccinia virus
	MSIMPVLTY	**MSIMPVL** A **Y**	42575	Vaccinia virus
	IAYRNDTSF	Y **YRNDTS** AI	434543	*Brucella abortus* 544
	KMRDTLPAK	**KMRDTLPAK**	84564	Vaccinia virus
	YVLSTIHIY	**YVLS** SL **HIY** W	91038	Vaccinia virus
	RSANMSAPF	**RSANTSAPF**	88180	Vaccinia virus
	KTFAIIAIV	**KTFAIIAIV**	33652	Vaccinia virus
B-Cell	STHRKVASSTTQYDHKESCN	S **STTQYDHKESCN** GLY	140072	Vaccinia virus
	YVHIGPLTKDKEDKVKKRYP	**KVKKRYPEF**	84867	Vaccinia virus
	VNTGPGGLSALLRQSYNGTA	**TGPGGLSALL**	63930	Vaccinia virus
	VHWNKKKYSSYEEAKKHDDG	INL **VHWNKKKYSSYEEAKKH**	102535	Vaccinia virus
	YFMKWLSDLREACFSYYQKY	ATTSPAREN **YFMRWLSDLRE**	102323	Vaccinia virus
	VYWYLGVNNLPYNWKNFYPS	**GVNNLPYNWK**	23074	Vaccinia virus
HTL	LIVIIYVFKKIKMNS	STL **IVTIYV**	61723	Vaccinia virus
	FGVYSILTSRGGITE	**S** V **LTSRGGI**	62237	Vaccinia virus
	VEVRYIDITNILGGV	**DITNILGGV** L	8802	Vaccinia virus
	MNFIPIIYSKAGKIL	**FIPIIYSKA**	16268	*Brucella abortus* 544 (Taxonomic Child)
	SPINIETKKAISDTR	**SPVNIE** AEPP	150593	Dengue virus
	IRDQYITALNHLVLS	**YITALNHLV**	74388	Vaccinia virus
	SLPYKYLQVVKQRER	LI **LPYK** F **L** NYIWI **Q** YKP	558705	Human beta herpes virus 6B

Underlined and bold letters are the similar fragments of peptides; Source organism of epitopes and the respective IEDB ID is also provided.

### Multiple sequence alignment

Final candidates seven B-cell epitopes show 100% sequence conservation with their source proteins across the selected MPXV isolates. Only STHRKVASSTTQYDHKESCN of the reference genome is mutated to STHRKVVSSTTQYDHKESCN in all chosen isolates (**Figure S1**). Similarly, all finalized CTL epitopes sequences are entirely conserved across the selected isolates (**Figure S2**), while only SPINIETKKAISDTR is mutated to SPINIETKKAISDAR amongst HTL epitopes (**Figure S3**). This suggests that the selected epitopes when use for developing multivalent epitope vaccine may offer broad therapeutic options for different MPXV variants.

### The conception of the multi-epitope vaccine for MPXV

**Figure 2:**
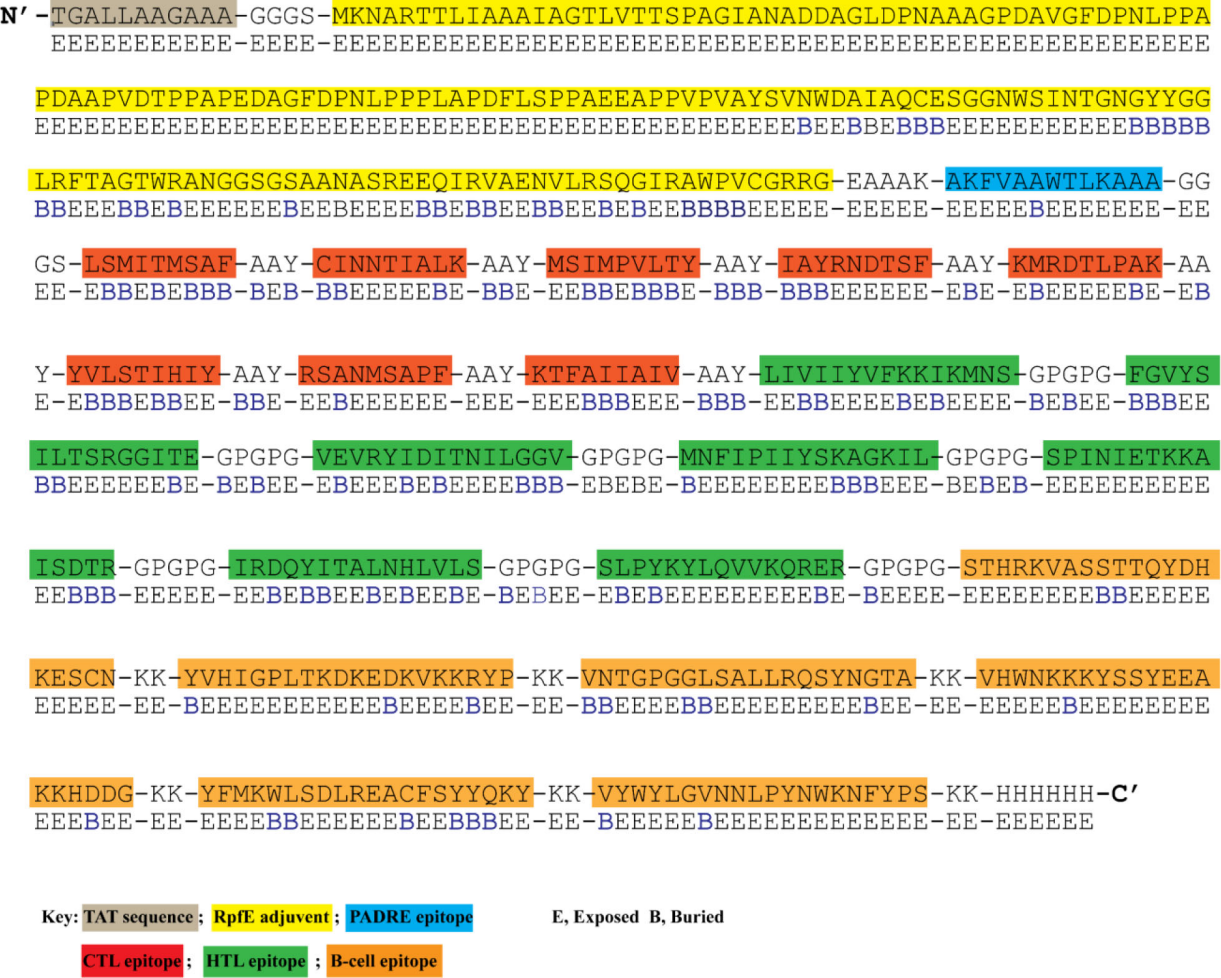
The designed vaccine’s amino acid sequence and surface accessibility. N-terminal has HIV TAT sequence attached. RpFE adjuvant sequence is highlighted in yellow color. PADRE epitope is shown in dark blue color. CTL, HTL, and B-cell epitopes are highlighted in red, green, and orange. Surface accessibility (NetSurfP - 3.0 sever) of each amino acid is shown as either exposed **(E)** or buried **(B)** residue.

To construct a multi-epitope vaccine, the final candidate epitopes were fused with the aid of appropriate linkers and an adjuvant. The final 583 amino acid long sequence of the constructed vaccine is presented in **Figure 2**. At the N-terminal, an HIV TAT (13aa) sequence was appended. A RpfE (172 aa) adjuvant was linked to the TAT sequence using the GGGS linker and established a connection with the PADRE epitope (11 aa) *via* the EAAAK linker. GGGS linker was inserted between the first CTL epitope and the PADRE sequence. Eight CTL epitopes were attached using the AAY linker. The same linker was added between the last CTL and the first HTL epitope. Similarly, seven HTL epitopes were separated with a GPGPGP linker from the first B-cell epitope. The connection of six B-cell epitopes was assisted by the KK linker. Lastly, a 6His tail was attached at the C-terminal *via* the KK linker.

### Vaccine surface accessibility, proteasomal processing, and population coverage

**Figure 3:**
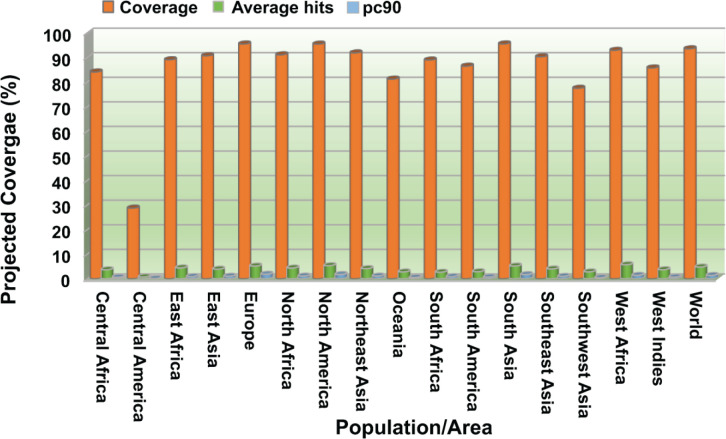
Population coverage analysis of combined CTL and HTL epitopes worldwide and across different regions. Coverage, projected population coverage; pc90, the minimum count of epitope hits/HLA combinations recognized by 90% of the population; Average hit/HLA combination, an average count of epitope hits/HLA combinations recognized by the population.

NetSurfP-3.0 sever returned the solvent accessibility of every residue in the multi-epitope vaccine ensemble (**Figure 2**). Such position (exposed [E]) are maximum described for 83.34% of residues within the B-cell epitopes, which suggested their accessibility for antibody recognition or B-cell receptor. In case of CD8+ T-cell epitopes, 61.16% of residues are exposed, and 38.86% are buried (B). For CD4+ T-cell epitopes, predominantly exposed residues (70.47%) are calculated. NetChop-3.1 server predicted 154 possible cleavage sites within the polypeptide vaccine sequence shown in **Table S1**. The estimated global population coverage of combined MHC-I and II epitopes is 93.62%, with a pc90 average of 1.41 and an average hit per HLA antigen of 4.83. The combined coverage of epitopes in South Asian, European, and North American populations is over 95%, while in Northeast Asia, North Africa, East Asia, and Southeast Asia, it is >90%. Apart from Southwest Asia (77.54%) and Central America (28.79%), the population coverage of collective epitopes in the other regions ranges from 81.29% to 89.16% (**Figure 3**).

### The multi-epitope vaccine accumulates features of a safety and efficacious antigen

Upon sequence similarity search, no significant match was found between the multi-epitope vaccine and Homo sapiens proteins. The chimeric epitopes were also dissimilar (based on<70% identity threshold) to human proteome when submitted individually to a BLASTp analysis. Thus, the final components of the chimeric vaccine were kept unchanged. As per AllerTOP v.2.0 and AllergenFP v.1.0, the designed vaccine is predicted as non-allergen. These results imply that the hypothetical antigen could possess safety in *in vivo* assays. Antigenicity testing of the vaccine indicated a score of 0.64 (VexiJen v2.0) and 0.87 (ANTIGENpro), categorizing the protein as a probable antigen. Besides, the Toxinpred server predicted the modelled vaccine as non-toxic (safe).

**Figure 4:**
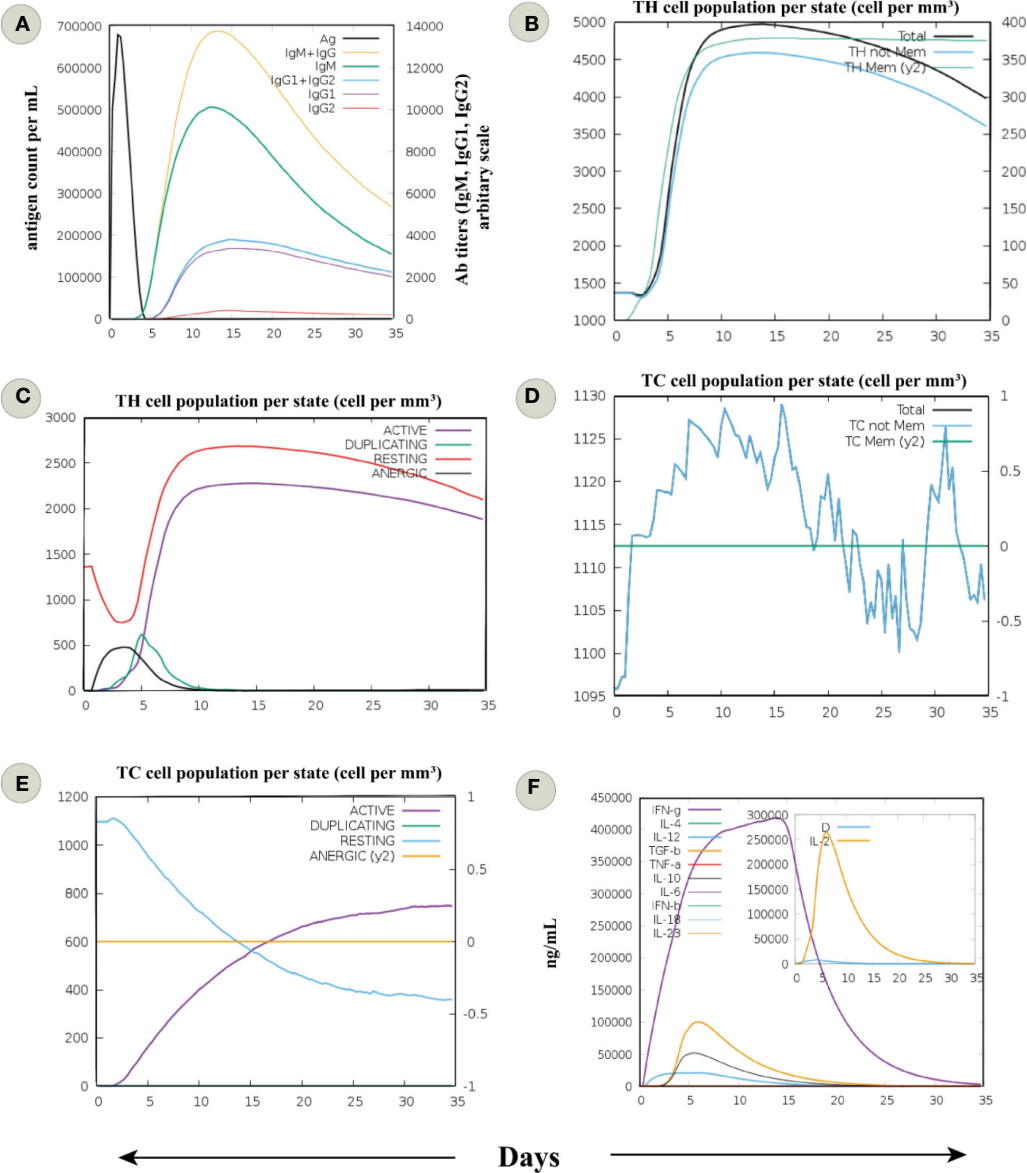
*In silico* immune simulation of an infection challenge, comprised of a virus responding to the sequence of MPXV proteins covered by the designed putative vaccine, was simulated for 35 days. **(A)** Immunoglobulin response against the antigen or vaccine. The development of immunoglobulin and immunocomplexes after the immunization signifies the induction of the humoral immune response with a change towards the diverse subtypes of immunoglobulins (immunoglobulin G (IgG), being more predominant). **(B)** The cell count of total and memory CD4+ T-helper (TH) lymphocytes. **(C)** The cell count of TH lymphocytes is shown in various forms, i.e., active, duplicating (in the mitotic cycle), resting (not active), and anergic. **(D)** The cell count of total and memory CD8+ T-cytotoxic (TC) lymphocytes. **(E)** The cell count of TC lymphocytes in various forms, i.e., active, duplicating (in the mitotic cycle), resting (not active), and anergic**. (F)** Levels of cytokines and interleukins. D in the inset plot is a danger signal and leukocyte growth factor IL-2.

*In silico* immune simulation showed behaviour consistent with the actual outcome upon vaccination; the secondary and tertiary one outweighed the primary immune response. Increased titers of IgM + IgG (>13000), IgM (>8000), IgG1 + IgG2, and IgG1 (>2000) antibodies and a subsequent reduction in antigen concentration was detected (**Figure 4A**). There was a rise in the active B-cell population following the fifth day of vaccine injection, and the total count remained persistent (>450 cells per mm^3^) till the end (**Figure S4A**). Total memory B-cell count with over 450 cells per mm^3^ was elevated, and IgM isotype level remained persistent throughout the simulation (**Figure S4B**). In case of plasma B-cell population, a peak level of isotype IgM+IgG level (>10 cells per mm^3^) was observed during 5–10 day period, followed by IgM (>8 cells per mm^3^) and IgG1 (>2 cells per mm^3^) (**Figure S4C**).

Comparable behaviour was seen for T helper (TH) cells, with a higher total count reaching 5000 cells per mm^3^ between 10–15 days. Besides, a constant count of memory TH cells (>4500 cells per mm^3^) was seen after the fifth day (**Figure 4B**). Also, many active TH cells were sustained until the end (**Figure 4C**). Accordingly, a significant concentration of memory T cytotoxic cells (TC) suggests the formation of a long-lasting cellular response (**Figure 4D**). The active TC cell count increased following the chimeric vaccine injection, with a peak value of over 600 cells per mm^3^ after the 15^th^ day (**Figure 4E** **)**. In addition, many active regulatory T cells (TH) were evident from the immune simulation outcome (**Figure S4D**). The findings revealed sustained levels of dendritic cells (DCs, >140 cells per mm^3^) and macrophages (>150 cells per mm^3^) among the innate immune cells (**Figures S4E, F**). Finally, levels of IFN-γ (>400000 ng/ml) and transforming growth factor-b (>50000 ng/ml) were substantially increased, and a higher Simpson index also indicated the generation of diverse cytokines in response to multi-epitope vaccine antigen (**Figure 4F**). These data signify the potential chimeric vaccine to induce long-lasting cellular and humoral immune response. However, an experimental evaluation of the proposed vaccine construct is recommended to clarify its ability to provoke adaptive immunity against MPXV.

### Physicochemical evaluation indicated encouraging parameters for vaccine manufacturing

Using the ProtParam server, a set of physicochemical parameters were computed for the designed vaccine construct. The estimated molecular weight of the construct is 62.29kDa, and the theoretical isoelectric point (pI) is 9.58 (alkaline). There are 39 and 66 negatively charged (Asp + Glu) and positively charged (Arg + Lys) residues, respectively. The estimated instability index of the multi-epitope vaccine is 39.37, which categorizes the protein as stable (a value >40 indicates a stable protein). Besides, a high aliphatic index of 74.63 reinforces protein stability in a wide range of temperatures. A long half-life period is crucial for the protein heterologous expression in bacteria or yeast. In this context, an estimated half-life of 7.2 h in mammalian reticulocytes (*in vitro*), over 20 h and 10 h in yeast, and *E. coli* (both *in vivo*), respectively, is obtained for the constructed vaccine. The solubility of the designed vaccine is supported by the SOLpro (0.53) and Protein-Sol (0.47) prediction. A negative GRAVY score (–0.27) also confirms the hydrophilic nature of the vaccine protein. Furthermore, no TM helices are predicted in the putative antigen, highlighting its suitability for future application.

### The designed vaccine attains a preferred 3D structure

The RoseTTAFold server yielded five potential 3D structures of the constructed vaccine. A model with more residues positioned in the favored regions of the Ramachandran plot and least in the disallowed regions was considered ideal. Model 2 was deemed the best among the predicted models because it contained 92.1% residues in the favored regions and 97.4% residues in the allowed regions of the Ramachandran plot. This model was submitted to the GlaxyRefine server, which also yielded five optimized models (**Table S2**). The refined Model 1 revealed more residues allocated to the favored region (94.7%) compared to the other models. Moreover, 98.8% of all residues were in the allowed regions, and only 1.2% were in the disallowed regions for this model. ProSA-web calculated Z-score of the initial and refined model was –9.33 and –9.14, respectively. A lower Z-score indicates that the refined model is the high-quality one. Z-score and Ramachandran plot details of the initial and refined model are provided in **Figure S5**. The 3D structural illustration of the refined modelled chimeric construct is presented in **Figure S5**.

### Chimeric vaccine folding forms discontinuous B-cell epitopes

The Elipro server foresaw a total of 307 residues distributed across 10 discontinuous/confirmational B-cell epitopes with scores ranging from 0.51 to 0.80. The size of epitopes ranged from four residues to 76 residues. The amino acid sequence and 3D structural depiction of the predicted conformational B-cell epitopes are shown in **Figure S6**.

### Molecular docking of T-cell epitopes with HLA receptors

**Figure 5:**
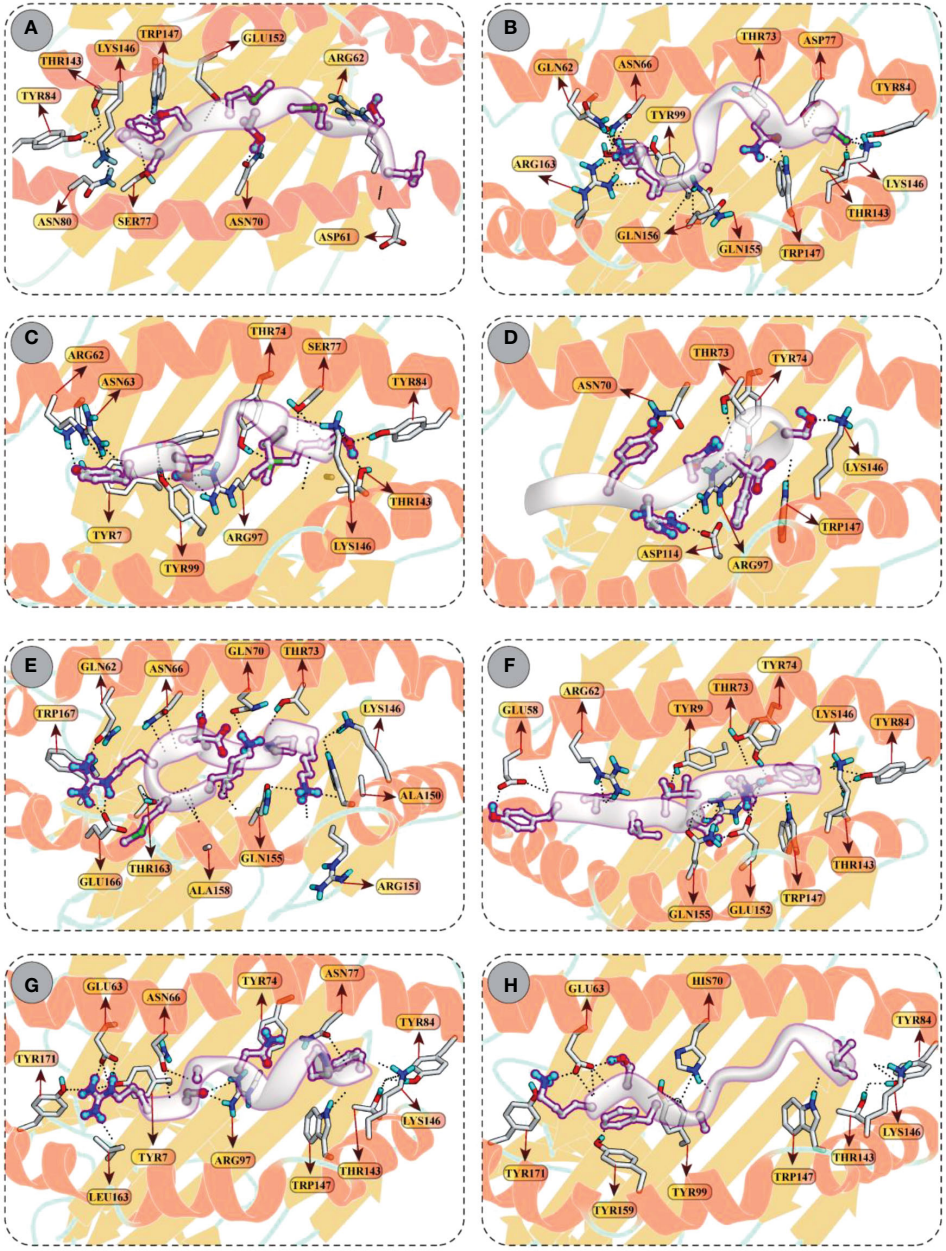
Molecular docking of CTL peptides (grey) and HLA class I molecules **(A)** Peptide LSMITMSAF attached with the binding groove of the HLA-B*15:01 **(B)** Peptide CINNTIALK attached with the binding groove of the HLA-A*11:01 **(C)** Peptide MSIMPVLTY attached with the binding groove of the HLA–B*35:01 **(D)** Peptide IAYRNDTSF attached with the binding groove of the HLA–B*35:01 **(E)** Peptide KMRDTLPAK attached with the binding groove of the HLA–A*30:01 **(F)** Peptide YVLSTIHIYV attached with the binding groove of the HLA-B*15:02 **(G)** Peptide RSANMSAPF attached with the binding groove of the HLA-B*58:01 **(H)** Peptide KTFAIIAIV attached with the binding groove of the HLA-A*02:06. All H-bonds are represented in dotted lines and the interacting residues of HLA class I receptor is shown in stick.

Molecular docking between CD8+ T-cell peptides and selected HLA molecules demonstrated DSs and several molecular interactions, including hydrogen bonding and ionic interactions (**Figure 5** and **Table S3**). The highest DS (–12.30 kcal/mol) was observed for KMRDTLPAK and RSANMSAPF with HLA-A*30:01 and HLA-B*58:01, respectively, followed by YVLSTIHIY with HLA-B*15:02 (–12.18 kcal/mol) and IAYRNDTSF with HLA-B*35:01 (–11.88 kcal/mol). In addition, LSMITMSAF, CINNTIALK, MSIMPVLTY, and KTFAIIAIV showed favourable binding with the binding groove of HLA-B*1501 (DS –11.34 kcal/mol), HLA-B*35:01 (DS –10.98 kcal/mol), HLA-A*02:06 (DS –10.61 kcal/mol), and HLA-A*11:01 (DS –10.17 kcal/mol), respectively.

**Figure 6:**
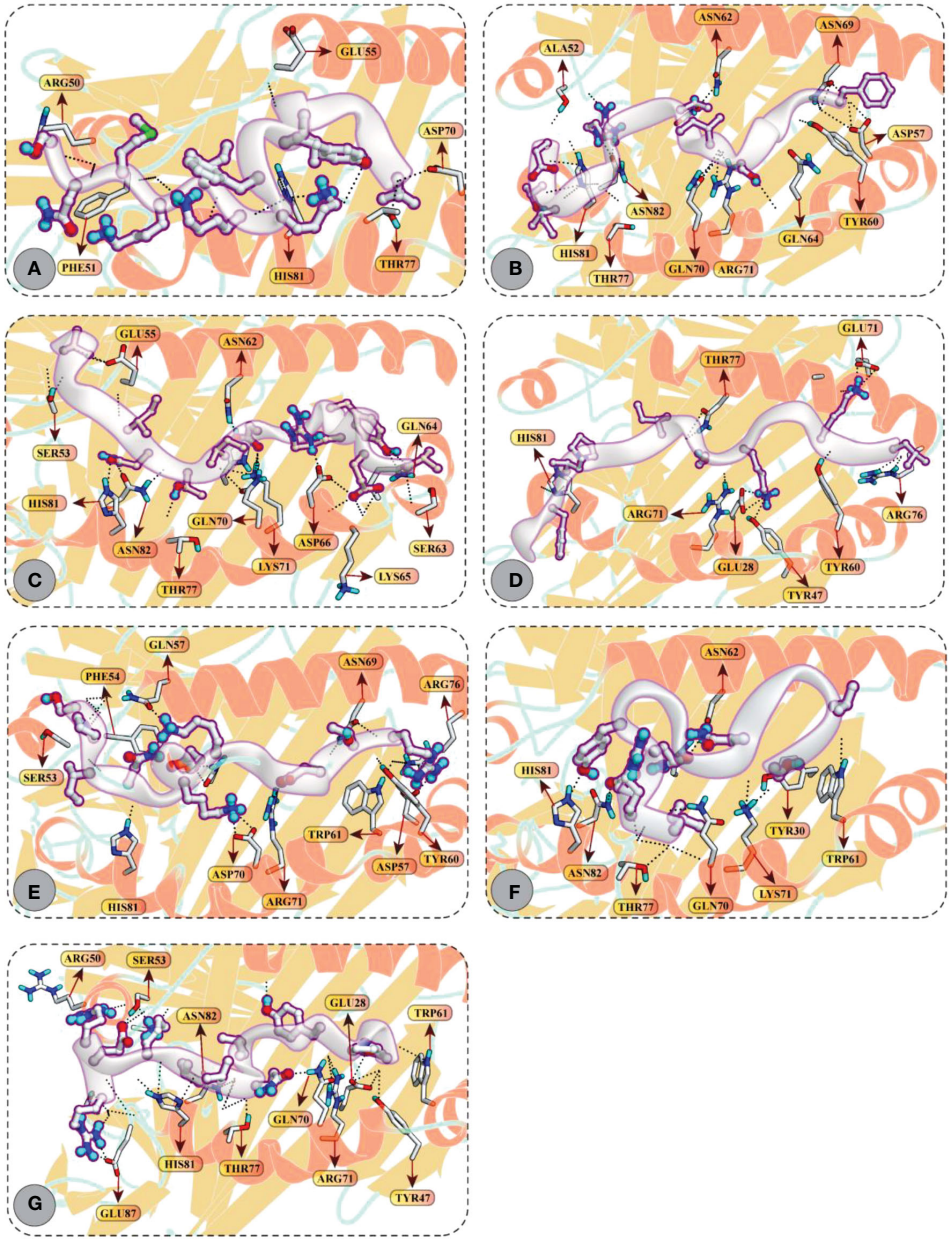
Molecular docking of HTL peptides and HLA class II molecules **(A)** Peptide LIVIIYVFKKIKMNS attached with the binding pocket of HLA-DRB1*11:01 **(B)** Peptide FGVYSILTSRGGITE attached with the binding pocket of HLA-DRB1*01:01 **(C)** Peptide VEVRYIDITNILGGV attached with the binding pocket of HLA-DRB1*04:01 **(D)** Peptide MNFIPIIYSKAGKIL attached with the binding pocket of HLA-DRB1*01:01 **(E)** Peptide SPINIETKKAISDTR attached with the binding pocket of HLA-DRB1*11:01 **(F)** Peptide IRDQYITALNHLVLS attached with the binding pocket of HLA-DRB1*04:01 **(G)** Peptide SLPYKYLQVVKQRER attached with the binding pocket of HLA-DRB1*01:01. All H-bonds are represented in dotted lines and the interacting residues of HLA class II molecule is shown in stick.

Accordingly, CD4+ T-cell peptides fit well into the binding groove of HLA-DR molecules with good DSs. For example, FGVYSILTSRGGITE (–12.97 kcal/mol), MNFIPIIYSKAGKIL (–12.35 kcal/mol), and SLPYKYLQVVKQRER (–12.41 kcal/mol) mediated good docking affinity with HLA-DRB1*01:01 molecule; IRDQYITALNHLVLS (–12.85 kcal/mol) and VEVRYIDITNILGGV (–11.92 kcal/mol) with HLA-DRB1*04:01; and LIVIIYVFKKIKMNS (–12.58 kcal/mol) and SPINIETKKAISDTR (–11.81 kcal/mol) with HLA-DRB1*11:01 molecule. All these epitopes mediated multiple hydrogen bonding and ionic interactions with the respective HLA receptors. All docking and molecular interaction details of CD4+ T-cell peptides and HLA-DR molecules are reported in **Figure 6** and **Table S4**.

### Molecular docking of the putative vaccine with TLR4

**Figure 7:**
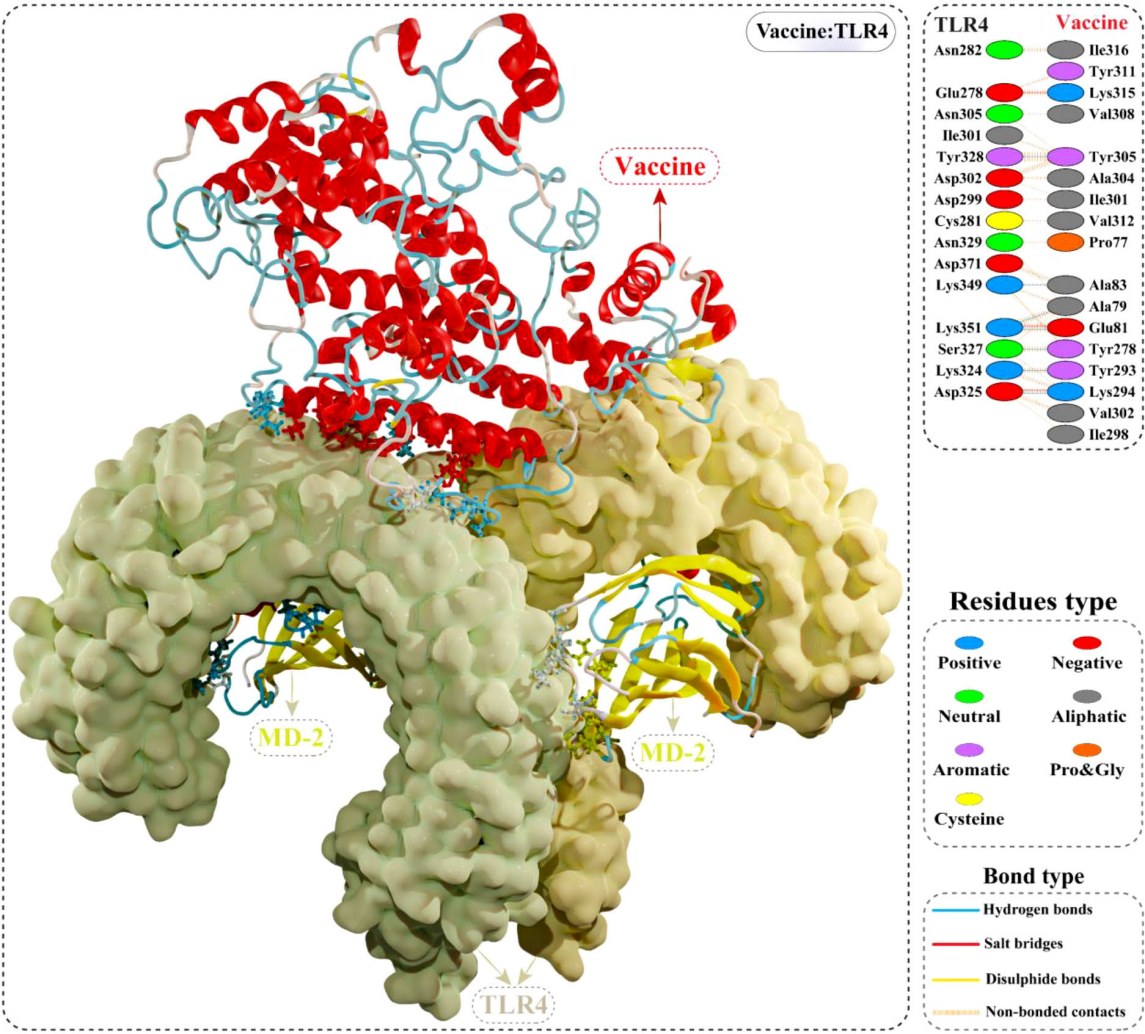
Optimal binding mode of the modelled vaccine (red) with TLR4 dimer (light yellow) obtained *via* molecular docking. Atomic interactions between the putative vaccine–TLR4 interface are shown at the top right. Hydrogen bonds are depicted in blue lines. The residue type is shown with a distinctive colour.

The cellular transport of an antigen molecule (putative vaccine in this case) and activation of appropriate downstream immune pathways would require interaction with the immune receptor (TLR). In this context, we performed molecular docking of multi-epitope vaccine with TLR4 to evaluate the binding energy and explore intermolecular contacts. The optimal-docked confirmation of the vaccine construct–TLR4 complex had a striking docking score of –79.80 kcal/mol, indicating the putative vaccine’s tight binding within the receptor binding pocket. Interface contact analysis of vaccine construct–TLR4 complex demonstrated strong intermolecular interactions, such as H-bonds, salt-bridge interactions, and non-bonded contacts. A total of seven residues of the constructed vaccine [Ala79, Glu81, Ala83, Tyr293, Lys294, Tyr278, and Tyr305] formed H-bonds with TLR4 residues with a bond distance in the range of 2.71Å to 2.82Å. Furthermore, Glu81, Lys294, and Lys315 residues of the modelled vaccine showed slat-bridges interactions with TLR4 within 2.73Å bond distance. Structural illustration and the detailed interface contacts between the modelled vaccine and TLR4 are shown in **Figure 7** and **Table S5**.

### Molecular dynamics simulations

**Figure 8:**
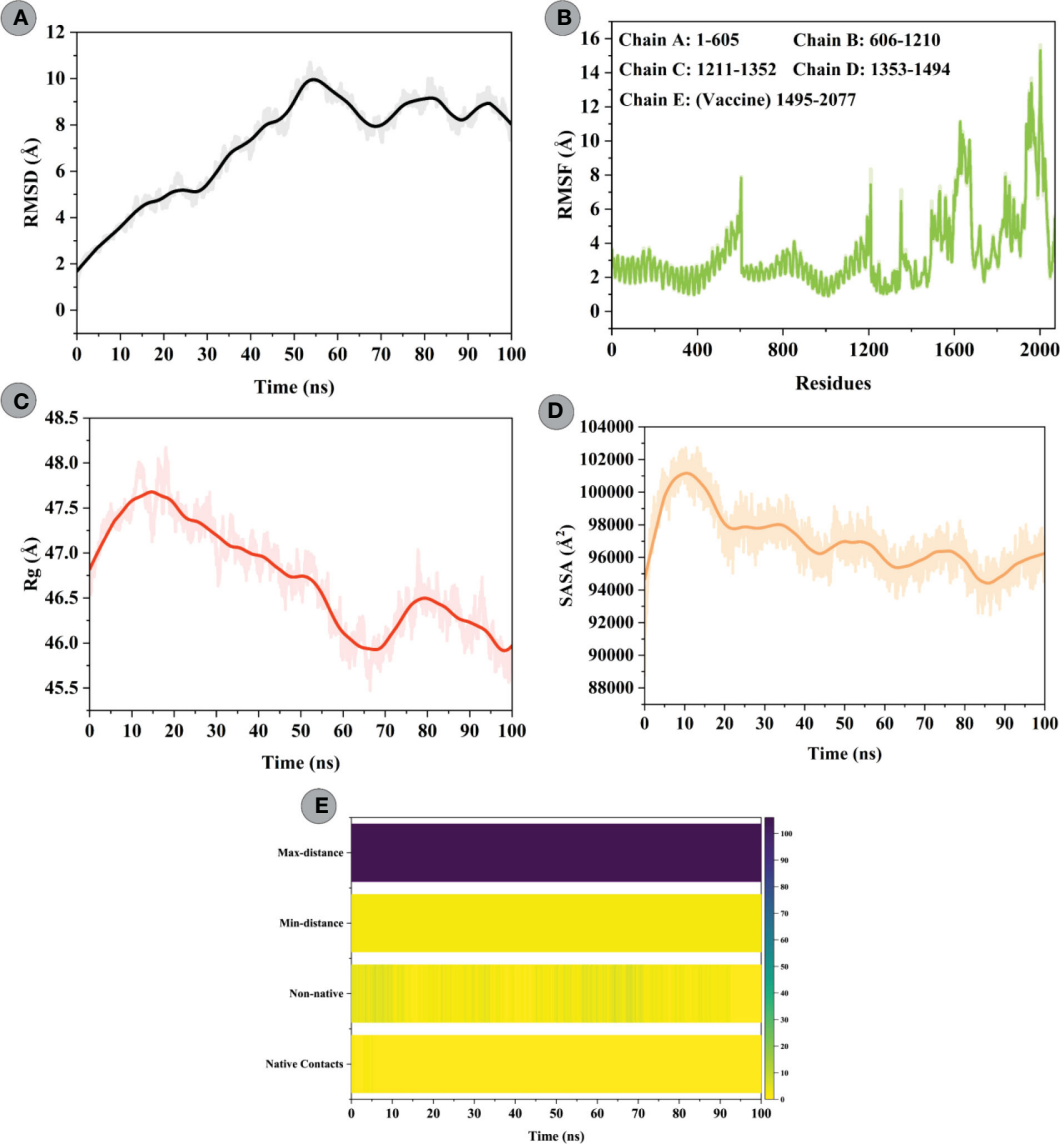
Molecular dynamics simulations study of modelled vaccine (ligand) and TLR4 (receptor) complex. **(A)** Root Mean Square Deviation (RMSD) plot of ligand and receptor complex present no significant displacement, indicating stable molecular interaction between two molecules. **(B)** Root Mean Square Fluctuation (RMSF) plot of the ligand-receptor complex representing the mobility of individual amino acid side chains. Amino acids of TLR4 goes from 1 to 1494, while that of vaccine construct starts from 1495 till the end. **(C)** The radius of Gyration (Rg) plot of ligand-receptor complex showing the structural compactness over 100ns timescale. **(D)** Solvent Accessible Surface Area (SASA) plot of ligand-receptor complex indicating changes in the surface volume of the complex over time (100ns). **(E)** Native and non-native contacts between the modelled vaccine–TLR4 complex over the simulation timescale.

To characterize the confirmational stability of constructed vaccine and TLR4 complex, an RMSD *vs*. time plot was analyzed. The average RMSD obtained for the modelled vaccine–TLR4 complex was 7.10Å. An increasing trend in RMSD was seen for this system till 55ns (starting from 1.63Å to peak RMSD of 9.93Å). After that, the system underwent slight fluctuations and attained stability towards the end (**Figure 8A**). RMSF was used to evaluate the flexibility of residues in the designed vaccine–TLR4 complex (**Figure 8B**). The mean RMSF of the vaccine–TLR4 complex was 3.40Å; the highest fluctuating residue was Thr508 (14.14; modelled vaccine residue), while the least mobile residue was Gly384 (0.97Å; TLR4 residue). The amino acid residues of the modelled vaccine that interacted with the TLR4 showed an RMSF<4Å. The analysis revealed that residues comprising adjuvant, HTL, and B-cell epitopes experienced greater flexibility which seems crucial for the modelled vaccine to attain favourable confirmation for binding immune cells. Structural compactness and regular packing of secondary structure elements (alpha-helix and beta-sheets) in the tertiary structure of protein was examined by plotting the Rg profile of modelled vaccine–TLR4 complex (**Figure 8C**). The average Rg of 46.73Å was computed for this complex. After hitting the peak of 47.73Å at 15ns, the Rg value steadily decreased to 45.71Å till 65ns. The value raised to 46.52Å at 78ns and gradually declined to 45.71Å before gaining equilibrium towards the end. The Rg plot of this complex indicated good compactness of the complex’s tertiary structure. Moreover, changes in the solvent accessibility of the constructed vaccine and TLR4 complex during the simulation was studied by performing the SASA analysis, which yielded a mean value of 97018.86Å². In the first 10ns, more residues were exposed towards the solvent side, whereby the SASA value increased from 94667.30Å² to 101161.17Å². Following that, the value gradually declined to 94432.12Å² till 80ns and then steadily improved till the end (**Figure 8D**). To inquire about the strength of intermolecular interactions across the simulation, we computed the pattern of H-bonds for the vaccine construct–TLR4 in every frame, applying a distance cut-off of 3Å. H-bond between Phe314^construct^ and Glu252^TLR4^ had the highest retention frequency of 77% during the simulation time. Pro78 and Ile293 residues of the vaccine construct showed H-bond with Leu325 and Asp299 of the TLR4, which were maintained for 12% and 11% of the simulation fraction, respectively. In addition, H-bond between Pro80 ^construct^ and Lys325^TLR4^, Ala304^construct^ and Tyr302^TLR4^, and Asn285^construct^ and Arg901^TLR4^ had retention a frequency of >5% during the simulation time. The detailed H-bond interactions with percent occupancy between modelled vaccine–TLR4 interface are enlisted in **Table S6**. Apart from native contacts, non-native contacts between the chimeric construct and TLR4 interface stayed persistent during the course of the simulation, suggesting stable binding of molecules (**Figure 8E**).

### Binding free calculation of constructed vaccine-TLR4 complex

The MM-GBSA calculation revealed the total bonding energy (ΔG Total) of the vaccine construct–TLR4 complex as –82.85 kcal/mol (**Table 6**). The high negative ΔG value points towards the energetically favourable binding and stability of the complex within the biological system. The dominant components of the interaction energy that contributed to the binding of the vaccine construct–TLR4 complex were electrostatic energy ΔE_elec_ (–2693.13 kcal/mol), gas-phase free energy ΔG_gas_ (–2553.01 kcal/mol), Van der walls energy ΔE_vdW_ (–283.87 kcal/mol), and the nonpolar component of the solvation energy Δ_ESURF_ (–29.92 kcal/mol).

**Table 6:** MM/GBSA free energy calculations and individual free energy components of the constructed vaccine TLR4 complex. Differences (Complex - Receptor - Ligand).

Energy Component	Vaccine-TLR4 complex (kcal/mol)	
	Average	Std. Err. of Mean
ΔE_vdW_	–283.87	0.33
ΔE_elec_	–2693.13	1.37
ΔE_GB_	2924.08	1.30
ΔE_SURF_	–29.92	0.03
ΔG _gas_	–2553.01	2.81
ΔG _solvation_	2470.16	1.78
ΔG _Total_	–82.85	0.04

ΔEvdw, van der Waals energy: ΔEEel, electrostatic energy: ΔEGB, polar component of solvation free energy (ΔGsolv): ΔEsurf, the nonpolar component of the solvation energy: ΔG gas, gas-phase free energy; ΔGTotal, total binding free energy.

### *In silico* cloning and prediction of RNA secondary structure

Prior to cloning, restriction sites of XhoI (CTC GAG at the N-terminal) and NheI (GCT AGC at the C-terminal) were added to flank the construct’s DNA sequence. The total length of the new DNA sequence was 1755 nucleotides (1743 nts. without restriction sites) and is presented in **Table S7** and **Figure S7**. Next, the nucleotide sequence was inserted into the pET-28a (+) expression plasmid between XhoI (site 158) and NheI (site 1907) by using the SnapGene software. The cloned plasmid with the designed chimeric vaccine had a total length of 7045 bp (**Figure S8**). The mRNA secondary structure prediction using the RNAfold program yielded optimal secondary structure and centroid secondary structure with a minimum free energy of –728.40 kcal/mol and –637.89 kcal/mol, respectively (**Figure S9**).

## Discussion

Historically, the development of vaccines has been thought the best cost-effective strategy for preventing illnesses resulting from infectious pathogens (67). Due to the possibility of combining potentially immunogenic components in a single construct, the multivalent or chimeric antigens constitute an attractive vaccine strategy (68, 69). Using various *in silico* and immunoinformatics approaches, researchers have designed such vaccines against several pathogens—Zika virus, Respiratory syncytial virus, Dengue virus, Leishmania donovani, *Mycobacterium Tuberculosis*, etc. (70–74). Yu et al. (75) developed an *in silico* multivalent vaccine model for SARS-CoV-2 and proved its immunogenicity through *in vitro* and *in vivo* experiments. Also, the multi-epitope vaccine was shown to develop protective efficacy *in vivo* (76, 77), and some of these vaccines have reached the clinical trial phase (78–80).

Naturally, surface proteins of the pathogens are more likely to come in contact with the host’s immune system and can evoke an immune response (81). Previously, the vaccinia virus membrane proteins have been evaluated as part of the subunit vaccine and target of neutralizing antibodies at *in vitro* and *in vivo* level (82). Recent immunoinformatics studies (83, 84) focused on E8L, B6R, and SERP2 proteins of MPXV to predict potential T-cell and B-cell epitopes and design multi-peptide-based vaccination capable of eliciting strong CD4+ and CD8+ T-cell associated immune response. Nevertheless, other proteins of MPXV with an exposed location could also be potential candidates for vaccine design. In the present report, we analyzed all membrane-bound, enveloped, and extracellular proteins of MPXV and selected 11 proteins following a reverse vaccinology pipeline to identify immunodominant T-cell and B-cell epitopes and develop a multi-epitope vaccine that can cross-protect against various strains of the virus responsible for the present outbreak. The chosen T-cell epitopes presented extensive coverage of the global population (>97% for MHC-I (81) and >95% for MHC-II (85). This feature is crucial for vaccine strategy, given the relevance of B-cells in antibody production and support of CTLs and HTLs in developing prolonged adaptive immunity against viral infection (86–88).

The immunogenic potential of the selected epitope sequences is revealed by their prediction as possible antigens. Induction of allergenicity is one of the significant barriers in vaccine development that occurs when a vaccine drives an immune response into an allergic reaction (89). Therefore, we chose epitopes predicted as non-allergen for the human immune system. Another critical factor in finalizing the epitopes was non-homology to proteins in the human proteome. No research reports the minimum percent identity that can prevent cross-reactivity between recognition of self-peptide and epitopes. That is why we kept the percent identity threshold of 70%, following Michel-Todó et al. (90), to limit the selection of MPXV epitopes similar to human peptides. For the B-cell and CD4+ T-cell epitopes, applying a 70% threshold is defined as conservative by a strategy intended to reduce the redundancy of epitopes. In case of CD8+ T-cell epitopes, position 2 and 9 is crucial for MHC-I molecule recognition, while residues 3–6 and 8 are involved in T-cell receptor (TCR) motif recognition. Applying this threshold could limit the selection of peptide that is similar to the human peptide of the same length in more than six residues (out of nine). Nevertheless, any potential cross-recognition may still occur with minimal similarity; thus, any cross-reactivity must be adequately managed (90). We expect that the bioinformatics tools will soon be able to predict this with more accuracy as the structural understanding of epitope recognition improves. To ensure that the CD8+ T-cell epitopes would differ in two critical positions from the human peptide, we could apply a 60% sequence identity threshold. By doing so, we would have obtained a single CTL (instead of eight), three HTL (instead of seven), and five B-cell (instead of six) epitopes. This would have lowered the number of T-cell epitopes that constitute the multi-epitope vaccine construct and could compromise the induction of robust cellular immune response. Thus, we opted to keep a 70% threshold to choose several epitopes for vaccine construction with suitable properties for production.

The genomes of MPXV and variola virus (an etiological agent of smallpox) share 96% similarity in the central regions and 83.5% to 93.6% similarity in the terminal regions—where most virulence and host-range coding genes are located (91). Lately, Ahmed et al. (13) found a mean genetic similarity of ~84% between MPXV-2022 sequences (MPXV genome isolates associated with the present outbreak) and reference vaccinia virus (GenBank: NC_006998.1). Previously, VACV vaccinia virus-based vaccine helped eradicate smallpox disease (13, 91). Besides, a first-generation VACV-based vaccine (Dryvax) has been observed to elicit cross-reactive immunity against the MPXV-CB upon immunization (13). In this work, most peptides used for the vaccine construction share 70% to 100% sequence conservation with the experimentally validated immune epitopes of VACV. Thus, based on the conspicuous resemblance between these viruses, we anticipate that the potential vaccine construct designed herein is expected to induce a cellular immune response against MPXV, similar to what is observed for the first-generation vaccines against MPXV-CB. Furthermore, because the reference VACV genome is ~98% identical to the VACV-derived vaccine sequences (Dryvax, ACAM2000, and MVA-BN), the cross-reactive immunity of these vaccines against newly emerged MPXVs is also anticipated (13). Additionally, sequence conservation of selected epitopes among the MPXV strains suggest that the potential vaccine construct can promote cross-protection among them.

The main benefit of using linkers in the context of multiple-epitope vaccine is that they prevent the formation of junctional antigens and improve the processing as well as presentation of antigens (92). CTL-HTL-B-cell epitopes were ordered in the putative vaccine construct in the same way as described in (93) and (94). In addition, considering (75, 93, 95, 96), we used various linkers: GGGS, EAAAK, AAY, GPGPG, and KK. To provide structural rigidity, an EAAAK linker was used that can reduce the hindrance of other protein’s regions during the interaction of the adjuvant and its receptor (81). Alternatively, the GGGGS linker was added to the construct to provide flexibility in the 3D structure (81). Other linkers were used mainly due to their ability to act as proteasomal cleavage site (AAY), induce HTLs response (GPGPG), and maintain the B-cell immunogenicity (KK) whilst bringing the pH closer to the physiological scale (70, 81). RpfE (a TLR4 agonist) was added as a natural adjuvant to increase the immunogenicity of the putative vaccine construct. Besides, using the TLR agonist as an adjuvant can increase the processing of antigens by the antigen-presenting cells (APCs) (97). RpfE interacts with the DCs and induces the differentiation of naïve CD+ 4 T-cells toward the Th4 and Th17 immune response. This reciprocal response evokes T-cells to secrete IFN-γ and IL-2 (97). The proteasomal cleavage predictor suggested that upon the cellular processing stage, the chimeric CTL epitopes would be generated; hence reinforcing that the distribution of selected linkers was appropriate.

The molecular weight of the designed chimeric antigen is less than 110kDa, which hinted its suitability for the wet-lab application. The computed half-life in *E. Coli* (7 h), instability index (39.37), and aliphatic index (74.63) of the chimeric vaccine construct supports its heterologous expression by using the *E. coli* system. For this reason, we carried out the codon adaptation and *in silico* cloning of multi-epitope vaccine in the common expression vector, pET28a (+). Moreover, the successful manufacturing of the modelled vaccine is supported by its solubility prediction. The immunological, physicochemical, and solubility properties of the designed chimeric construct remained consistent with its original version after incorporating the mutations (T>A at position 399 and A>V at position 452). Droppa-Almeida et al. (98) and Rekik et al. (99) modelled the tertiary structure of constructed vaccine with a Z-score of −5.26 kcal/mol, and −9.51 kcal/mol, respectively, and concluded that the predicted structure is of preferable quality. In the current study, the predicted tertiary structure of the chimeric construct had a Z-score of −9.11, corresponding to the X-Ray crystallographic determined structures of the proteins of similar sizes.

TLRs are the pattern recognition receptors (PRRs) expressed on innate immune (DCs and macrophages) and non-immune cells (fibroblast cells and epithelial cells). They play a central part in innate immunity by recognizing the conserved pathogen-associated molecular pattern (PAMP) derived from various microorganisms (100). TLR4 receptor (an extracellular TLR) has been reported to detect the structural proteins of different viruses and stimulate inflammatory cytokines’ secretion against them (101–103). We carried out P–P docking to estimate the designed vaccine’s affinity with TLR4. The docking calculation revealed stable binding (high negative S score) of the modelled vaccine with this receptor involving several intermolecular interactions (H-bonds, ionic and hydrophobic contacts). Correspondingly, the mutant version of the modelled vaccine (T>A at position 399 and A>V at position 452) displayed a high docking score (–94.52 kcal/mol) with TLR4. Coherently, RMSD and Rg descriptors indicated a stable interaction, while RMSF and SASA descriptors revealed structural alterations (which could be vital to attain a favourbale confirmation for appropriate exposure to the immune cells) when the modelled vaccine binds to TLR4. Besides, H-bond analysis indicated a persistent H-bond interaction between the modelled vaccine and immune receptor, which is vital for complex stability (104). MM-GBSA calculations also affirmed the high-affinity and energetically favourable binding (low –ΔG value) of the modelled vaccine and TLR4.

*In silico* immune simulation predicted high titers of neutralizing antibodies following immunization, which are crucial to fight the viral infection. Further, the presence of PADRE epitope in the polypeptide vaccine can promote anti-tumor response by expanding CTLs, HTLs, and IFN-γ production, as reported by Ghaffari-Nazari et al. (105). In our study, immune simulation outcomes predicted a higher level of IFN-γ and lifelong cellular responses. These data highlight the likelihood of the potential vaccine candidate provoking a robust immune response capable of protecting against MPX disease. Amongst the selected proteins, A35R, C4L, and E8L have been investigated as a subset of an antigen in subunit vaccines against MPX and smallpox (106–112). Altogether, the potential vaccine candidate’s immunogenic, physicochemical, and structural features suggest that it might produce promising results *in vitro* and *in vivo*.

## Limitations

To address the antigenic complexity, this study presented an alternate vaccine design strategy based on a multi-epitope ensemble of the antigenic proteins of the MPXV. There are some limitations to the current investigation. The designed vaccine is thought to be immunogenic after being evaluated using immunoinformatics methods; however, the accuracy of these methods are not perfect, and it is unclear how well the modelled vaccine will protect against MPX infection. In addition, immunoinformatics methods have various challenges, such as standard benchmarking, constrained prediction approaches, and a paucity of datasets for different computational investigations. In recent years, several successful cases have been documented (113); thus, the proposed multivalent epitope vaccine warrants investigation using *in vitro* and *in vivo* bioassays for experimental validation to demonstrate its safety based on the prediction findings.

## Conclusion

The present study implemented reverse vaccinology and immunoinformatics approaches to explore the membrane-bound enveloped and extracellular proteins of MPXV to design a broad-spectrum vaccine candidate to tackle the viral infection. Multiple immunodominant B-cell, CD4+, and CD8+ T-cell epitopes were predicted from the selected proteins that were assembled into an antigenic, non-allergen, and non-toxic multi-epitope-based vaccine construct. To increase the immune response, stability, and effectiveness of the engineered vaccine construct, various linkers and an adjuvant sequence were added to it. The collective population coverage of the selected epitopes was 93.62% globally and was conserved across different strains of MPXV, suggesting the potency of wider protection against several viral strains worldwide. Moreover, the higher similarity of selected epitopes with the experimentally validated vaccinia virus epitopes could facilitate the designed vaccine’s rapid advancement to the experimental stage. MD simulation and MM-GBSA analysis confirmed the stability and higher binding affinity (–82.85 kcal/mol) of the chimeric construct and TLR4 complex. Besides, immune stimulation revealed that humoral and cellular immune responses would be generated against the MPXV upon administration of such a potent multi-epitope vaccine. However, experimental validation of safety and efficacy and preclinical studies of the designed vaccine is required before human immunization.

## Data availability statement

The original contributions presented in the study are included in the article/**Supplementary Material**. Further inquiries can be directed to the corresponding authors.

## Author contributions

MW, SA and FA conceived and designed the study. SA, MW and YK performed experiments. AA, AI, and MW analyzed the data. PL, review the manuscript first draft. MW, SA, FA and FK wrote the manuscript with inputs and comments from all co-authors. All authors contributed to the article and approved the submitted version.
