# Supplementary material for: Immunoinformatics design of multivalent epitope vaccine against monkeypox virus and its variants using membrane-bound, enveloped, and extracellular proteins as targets
Source: Front Immunol. 2023 Jan 26;14:1091941. doi: 10.3389/fimmu.2023.1091941 (PMC9908764; doi:10.3389/fimmu.2023.1091941)
Supplement: Supplementary file 1 [file DataSheet_1.docx]

**Immunoinformatics Design of Multivalent Epitope Vaccine against Monkeypox Virus and its Variants Using** **Membrane-bound, Enveloped, and Extracellular Proteins as a Target**

Muhammad Waqas^1,2†^, Shahkaar Aziz^3†^, Pietro Liò^4^, Yumna Khan^3^, Amjad Ali^1*^, Aqib Iqbal^5*^, Faizullah Khan^2,6^, Fahad Nasser Almajhdi^7*^

^1^Department of Biotechnology and genetic Engineering, Hazara University, Mansehra 21120, Pakistan.

^2^Natural and Medical Sciences Research Center, University of Nizwa, Birkat-ul-Mouz 616, Nizwa, Oman.

^3^Institute of Biotechnology and Genetic Engineering, The University of Agriculture, Peshawar 25130, Pakistan.

^4^Department of Computer Science and Technology, University of Cambridge, Cambridge, United Kingdom

^5^Department of Biotechnology, Abdul Wali Khan University Mardan, Mardan 23200, Pakistan

^6^Department of Pharmacy, Abdul Wali Khan University Mardan, Mardan 23200, Pakistan

^7^Department of Botany and Microbiology, College of Science, King Saud University, Riyadh 11451, Saudi Arabia.

*** Correspondence:**

Amjad Ali

amjad.genetics@hu.edu.pk

Aqib Iqbal

Aqib72@aup.edu.pk

Fahad Nasser Almajhdi

majhdi@ksu.edu.sa

^†^ = These authors equally contributed

**Table of contents**

| **Table S1.** | Proteasomal processing of the constructed vaccine. Predictions were made using the NetChop server. Seq. sequence, Pred. Prediction, S cleavage site |
| --- | --- |
| **Table S2** | GalaxyRefine output for the submitted initial model of vaccine constructs predicted by the RoseTTAFold server. (Letter in bold indicates the final model selected for further analysis). |
| **Table S3** | Molecular docking calculations and interactions details between selected CTL epitopes and respective HLA class I allele (MOE2020). H, hydrogen bond; IH, Ionic interaction; A, Arene interaction |
| **Table S4*.*** | Molecular docking calculations and interactions details between selected HTL epitopes and respective HLA class II allele (MOE2020). H, hydrogen bond; IH, Ionic interaction; A, Arene interaction |
| **Table S5.** | Atom-atom interactions across TLR4–vaccine construct interface obtained using PDBsum server |
| **Table S6.** | Hydrogen bonds at the TLR4– vaccine construct interface (with occupancy ≥ 2%) |
| **Table S7.** | Complementary DNA sequence of the constructed monkeypox vaccines subjected to in-silico cloning and RNA structure prediction in this study. The N-terminal contains inserted XhoI restriction site, and the C-terminal contains inserted NheI restriction site (shown in bold letters). |
| **Figure S1.** | Multiple sequence alignment of candidates B-cell epitopes with selected monkeypox isolates using the Clustal Omega program. Only STHRKVASSTTQYDHKESCN of the reference genome is mutated to STHRKVVSSTTQYDHKESCN in all selected MPXV isolates. |
| **Figure S2.** | Multiple sequence alignment of candidates CTL epitopes with selected monkeypox isolates using the Clustal Omega program. All epitopes perfectly aligned with the same protein sequence from monkeypox isolates showing 100% sequence conservation. |
| **Figure S3.** | Multiple sequence alignment of candidates HTL epitopes with selected monkeypox isolates using the Clustal Omega program. Only SPINIETKKAISDTR is mutated to SPINIETKKAISDAR in selected monkeypox isolates. |
| **Figure S4.** | In silico immune simulation of an infection challenge, comprised of a virus responding to the sequence of MPXV proteins covered by the multi-epitope construct, was simulated on day 35. (A) B lymphocytes population per entity-state (i.e., showing counts for active, presenting on class II, internalized the Ag, duplicating and anergic (B) B lymphocytes: total count, memory cells, and sub-divided in isotypes IgM, IgG1 and IgG2 (C) Plasma B lymphocytes count sub-divided per isotype (IgM, IgG1 and IgG2) (D) CD4 T-regulatory lymphocytes count. Both total, memory and per entity-state counts are plotted here (E) Dendritic cells can present antigenic peptides on both MHC class-I and class-II molecules. The curves show the total number broken down to active, resting, internalized, and presenting the ag (F) Total count, internalized, presenting on MHC class-II, active and resting macrophages. |
| **Figure S5.** | Structure validation of the chimeric vaccine’s modeled tertiary structure. Primary modeled structure (before refinement) (A) Ramachandran plot details and (B) Z-score graph. Final modeled structure (after refinement), (C) Ramachandran plot details, and (D) Z-score graph. The Pro-SA web-generated Z-score graph shows the modeled 3D structure corresponding to the X-Ray crystallographic determined structure for the protein of similar sizes. (E) 3D structural illustration of the refined modeled vaccine construct. |
| **Figure S6.** | Discontinuous/ Confirmational B-cell epitopes are predicted within the designed vaccine (ElliPro server). The predicted server score is shown with each figure. |
| **Figure S7.** | Adapted cDNA construct of the designed vaccine (with restriction enzymes sites added) used for in silico cloning in the expression vector. |
| **Figure S8**. | Simulated cloning of the designed multi-epitope vaccine construct for MPXV. The adapted DNA sequence of the proposed construct (shown in red) was inserted into the pET-28a (+) expression vector between the XhoI and NheI enzyme loci. |
| **Figure S9.** | Predicted minimum free energy (MFE) mRNA secondary structure (A) and centroid mRNA secondary structure (B) of the designed vaccine (RNAfold program). The MFE structures are colored by base-pairing probabilities. For unpaired regions the color denotes the probability of being unpaired. |

**Table S1.** Proteasomal processing of the constructed vaccine. Predictions were made using the NetChop server. Seq. sequence, Pred. Prediction, S cleavage site

| Seq. | TGALLAAGAAAGGGSMKNARTTLIAAAIAGTLVTTSPAGIANADDAGLDPNAAAGPDAVGFDPNLPPAPDAAPVDTPPAP |
| --- | --- |
| Pred. | ...SS.S........SS..........S...SS......S..SS...S..........S.S.S.S....SSS.S....SS |
| Seq. | EDAGFDPNLPPPLAPDFLSPPAEEAPPVPVAYSVNWDAIAQCESGGNWSINTGNGYYGGLRFTAGTWRANGGSGSAANAS |
| Pred. | ..S.S.S.S...SS.SSS..SS.......SSS.S.S.SSS.........S.....SS..S.S....SSS......S.... |
| Seq. | REEQIRVAENVLRSQGIRAWPVCGRRGEAAAKAKFVAAWTLKAAAGGGSLSMITMSAF**AAY**CINNTIALK**AAY**MSIMPVL |
| Pred. | ......SS...SS......S.S..SS......S.....S.SSS.S......S....S...S..........SS....... |
| Seq. | TY**AAY**IAYRNDTSF**AAY**KMRDTLPAK**AAY**YVLSTIHIY**AAY**RSANMSAPF**AAY**KTFAIIAIV**AAY**LIVIIYVFKKIKMNS |
| Pred. | .S.SS........SSS.........S.S.........SS...........SS.S........S................ |
| Seq. | GPGPGFGVYSILTSRGGITEGPGPGVEVRYIDITNILGGVGPGPGMNFIPIIYSKAGKILGPGPGSPINIETKKAISDTR |
| Pred. | S.....................S.S. SS....S..........S.S.SS..SS.......S....SS.S.......... |
| Seq. | GPGPGIRDQYITALNHLVLSGPGPGSLPYKYLQVVKQRERGPGPGSTHRKVASSTTQYDHKESCNKKYVHIGPLTKDKED |
| Pred. | .........S.. SS..SSS.......S.S......S.S.S.........SSSS....S.S..S....SS......S.S. |
| Seq. | KVKKRYPKKVNTGPGGLSALLRQSYNGTAKKVHWNKKKYSSYEEAKKHDDGKKYFMKWLSDLREACFSYYQKYKKVYWYL |
| Pred. | . S.S.S.SSS......S.SSS...S... S.SS...S..SS.......SS.SS...S......SS..S..S.S...... |
| Seq. | GVNNLPYNWKNFYPSKKHHHHHH |
| Pred. | ............S..SS…S.S.. |

**Table S2.** GalaxyRefine output for the submitted initial model of vaccine constructs predicted by the RoseTTAFold server. (Letter in bold indicates the final model selected for further analysis).

| Model | GDT-HA | RMSD | MolProbity | Clash score | Poor rotamers | Rama favored |
| --- | --- | --- | --- | --- | --- | --- |
| Initial | 1.0000 | 0.000 | 1.586 | 3.1 | 0.0 | 92.1 |
| MODEL 1 | 0.9717 | 0.348 | 1.891 | 9.8 | 0.7 | 94.5 |
| MODEL 2 | 0.9798 | 0.325 | 1.867 | 9.7 | 0.5 | 94.8 |
| MODEL 3 | 0.9704 | 0.357 | 1.849 | 9.3 | 0.2 | 94.8 |
| MODEL 4 | 0.9760 | 0.330 | 1.921 | 10.6 | 0.7 | 94.5 |
| MODEL 5 | 0.9803 | 0.315 | 1.899 | 10.3 | 0.2 | 94.7 |

**Table S3.** Molecular docking calculations and interactions details between selected CTL epitopes and respective HLA class I allele (MOE2020). H, hydrogen bond; IH, Ionic interaction; A, Arene interaction

| Epitope | Receptor PDB-ID | Docking score (kcal/mol) | Bond Type | Receptor Residues | Epitopes Residues | Energy (kcal/mol) | Distance (Å) |
| --- | --- | --- | --- | --- | --- | --- | --- |
| LSMITMSAF | 3C9N | –11.34 | H | Asp61 | Leu1 | -3.80 | 2.73 |
|  |  |  | H | Arg62 | Ser2 | -2.00 | 3.18 |
|  |  |  | H | Arg62 | Met3 | -3.10 | 2.92 |
|  |  |  | H | Asn70 | Thr5 | -3.00 | 2.93 |
|  |  |  | H | Ser77 | Phe9 | -2.70 | 2.91 |
|  |  |  | H | Asn80 | Phe9 | -5.20 | 2.70 |
|  |  |  | H | Tyr84 | Phe9 | -5.20 | 2.69 |
|  |  |  | A | Leu95 | Phe9 | -1.30 | 4.16 |
|  |  |  | H | Thr143 | Phe9 | -3.20 | 2.62 |
|  |  |  | IH | Lys146 | Phe9 | -20.01 | 2.75 |
|  |  |  | H | Trp147 | Ala8 | -0.90 | 2.99 |
|  |  |  | H | Glu152 | Met6 | -7.80 | 2.76 |
|  |  |  | H | Glu152 | Ser7 | -2.60 | 3.11 |
| CINNTIALK | 5WJN | –10.17 | H | Asp61 | Leu1 | -3.80 | 2.73 |
|  |  |  | H | Arg62 | Ser2 | -2.00 | 3.18 |
|  |  |  | H | Arg62 | Met3 | -3.10 | 2.92 |
|  |  |  | H | Asn70 | Thr5 | -3.00 | 2.93 |
|  |  |  | H | Ser77 | Phe9 | -2.70 | 2.91 |
|  |  |  | H | Asn80 | Phe9 | -5.20 | 2.70 |
|  |  |  | H | Tyr84 | Phe9 | -5.20 | 2.69 |
|  |  |  | A | Leu95 | Phe9 | -1.30 | 4.16 |
|  |  |  | H | Thr143 | Phe9 | -3.20 | 2.62 |
|  |  |  | IH | Lys146 | Phe9 | -20.01 | 2.75 |
|  |  |  | H | Trp147 | Ala8 | -0.90 | 2.99 |
|  |  |  | H | Glu152 | Met6 | -7.80 | 2.76 |
|  |  |  | H | Glu152 | Ser7 | -2.60 | 3.11 |
| MSIMPVLTY | 4PR5 | –10.98 | H | Tyr9 | Thr8 | -4.00 | 2.74 |
|  |  |  | IH | Arg62 | Tyr9 | -33.86 | 2.80 |
|  |  |  | H | Tyr74 | Met1 | -2.40 | 3.05 |
|  |  |  | H | Ser77 | Ser2 | -2.00 | 3.07 |
|  |  |  | H | Ser77 | Ile3 | -2.80 | 2.86 |
|  |  |  | H | Tyr84 | Ser2 | -1.80 | 2.88 |
|  |  |  | H | Arg97 | Met1 | -0.70 | 3.74 |
|  |  |  | H | Arg97 | Thr8 | -6.30 | 2.95 |
|  |  |  | H | Ala117 | Met1 | -4.50 | 3.13 |
|  |  |  | H | Thr143 | Ser2 | -1.80 | 2.73 |
|  |  |  | H | Lys146 | Ser2 | -6.00 | 2.95 |
|  |  |  | H | Lys146 | Ile3 | -7.50 | 2.81 |
|  |  |  | H | Lys146 | Met4 | -1.20 | 4.04 |
|  |  |  | H | Trp147 | Ser2 | -2.50 | 2.81 |
| IAYRNDTSF | 4PR5 | –11.88 | H | Thr73 | Asp6 | -1.60 | 3.02 |
|  |  |  | H | Tyr74 | Phe9 | -2.70 | 2.57 |
|  |  |  | H | Arg97 | Asn5 | -6.50 | 2.81 |
|  |  |  | IH | Arg97 | Phe9 | -15.80 | 3.06 |
|  |  |  | IH | Asp114 | Arg4 | -13.76 | 3.13 |
|  |  |  | H | Lys146 | Ser8 | -6.70 | 2.86 |
|  |  |  | H | Trp147 | Ser8 | -2.90 | 2.98 |
|  |  |  | A | Trp147 | Phe9 | -0.00 | 3.60 |
|  |  |  | A | Trp167 | Ile1 | -0.50 | 3.33 |
| KMRDTLPAK | 6J1W | –12.30 | H | Gln62 | Arg3 | -6.00 | 2.83 |
|  |  |  | H | Thr73 | Pro7 | -1.90 | 2.71 |
|  |  |  | A | Tyr99 | Leu6 | -1.40 | 4.00 |
|  |  |  | IH | Lys146 | Lys9 | -29.02 | 2.91 |
|  |  |  | H | Trp147 | Lys9 | -3.80 | 2.76 |
|  |  |  | H | Ala150 | Lys9 | -13.30 | 2.72 |
|  |  |  | H | Arg151 | Lys9 | -5.90 | 2.77 |
|  |  |  | H | Gln155 | Lys1 | -11.70 | 3.05 |
|  |  |  | H | Gln155 | Lys9 | -0.80 | 3.12 |
|  |  |  | A | Tyr159 | Thr5 | -0.50 | 4.84 |
|  |  |  | H | Thr163 | Met2 | -0.70 | 2.71 |
|  |  |  | IH | Glu166 | Arg3 | -28.75 | 2.77 |
| YVLSTIHIY | 6UZM | –12.18 | H | Tyr9 | Ser4 | -1.40 | 2.89 |
|  |  |  | IH | Glu58 | Tyr1 | -23.60 | 2.72 |
|  |  |  | H | Arg62 | Val2 | -7.90 | 3.03 |
|  |  |  | H | Tyr84 | Tyr9 | -5.10 | 2.73 |
|  |  |  | H | Arg97 | Ser4 | -7.90 | 2.88 |
|  |  |  | H | Thr143 | Tyr9 | -3.20 | 2.63 |
|  |  |  | IH | Lys146 | Tyr9 | -21.88 | 2.71 |
|  |  |  | H | Trp147 | Ile8 | -3.60 | 2.73 |
|  |  |  | H | Glu152 | Thr5 | -1.90 | 2.58 |
|  |  |  | H | Glu152 | His7 | -7.10 | 3.05 |
|  |  |  | H | Glu152 | Ile8 | -0.50 | 3.47 |
|  |  |  | H | Gln155 | Ile6 | -6.40 | 2.91 |
| RSANMSAPF | 5VWH | –12.30 | IH | Glu63 | Arg1 | -26.70 | 3.04 |
|  |  |  | H | Asn66 | Ala3 | -2.50 | 2.83 |
|  |  |  | H | Tyr74 | Asn4 | -1.90 | 2.68 |
|  |  |  | A | Asn77 | Phe9 | -0.70 | 3.69 |
|  |  |  | H | Tyr84 | Phe9 | -4.00 | 2.58 |
|  |  |  | H | Arg97 | Ser2 | -1.80 | 2.84 |
|  |  |  | H | Thr143 | Phe9 | -3.80 | 2.66 |
|  |  |  | IH | Lys146 | Phe9 | -32.05 | 2.83 |
|  |  |  | A | Trp147 | Met5 | -0.80 | 3.67 |
|  |  |  | H | Trp147 | Pro8 | -1.80 | 2.83 |
|  |  |  | H | Leu163 | Arg1 | -4.10 | 2.93 |
| KTFAIIAIV | 3OXR | –10.61 | IH | Glu63 | Lys1 | -45.88 | 2.85 |
|  |  |  | H | Glu63 | Thr2 | -9.40 | 2.78 |
|  |  |  | A | Lys66 | Phe3 | -0.70 | 4.02 |
|  |  |  | H | His70 | Ala4 | -2.50 | 2.86 |
|  |  |  | H | Tyr84 | Val9 | -5.10 | 2.62 |
|  |  |  | H | Thr143 | Val9 | -3.20 | 2.61 |
|  |  |  | IH | Lys146 | Val9 | -21.48 | 3.02 |
|  |  |  | H | Trp147 | Ile8 | -4.40 | 2.80 |
|  |  |  | A | Trp167 | Lys1 | -1.30 | 3.56 |

**Table S4**. Molecular docking calculations and interactions details between selected HTL epitopes and respective HLA class II allele (MOE2020). H, hydrogen bond; IH, Ionic interaction; A, Arene interaction

| Epitope | Receptor PDB-ID | Docking score (kcal/mol) | Bond Type | Receptor Residues | Epitopes Residues | Energy (kcal/mol) | Distance (Å) |
| --- | --- | --- | --- | --- | --- | --- | --- |
| LIVIIYVFKKIKMNS | 6CPN | –12.58 | H | Phe51 | Met13 | -2.30 | 2.89 |
|  |  |  | A | Phe51 | Asn14 | -0.90 | 4.81 |
|  |  |  | H | Glu55 | Ile4 | -1.50 | 2.99 |
|  |  |  | IH | Asp70 | Leu1 | -21.16 | 3.09 |
|  |  |  | H | Asp76 | Lys9 | -1.60 | 3.28 |
|  |  |  | H | Thr77 | Leu1 | -6.80 | 2.87 |
|  |  |  | H | Thr77 | Lys9 | -7.80 | 2.81 |
|  |  |  | H | His81 | Lys9 | -0.70 | 3.39 |
|  |  |  | H | Gly84 | Ile11 | -1.90 | 2.98 |
| FGVYSILTSRGGITE | 1AQD | –12.97 | H | Gln9 | Thr8 | -0.90 | 2.95 |
|  |  |  | H | Ser53 | Arg10 | -5.50 | 2.78 |
|  |  |  | H | Ser53 | Glu15 | -2.60 | 2.59 |
|  |  |  | IH | Asp57 | Phe1 | -16.00 | 2.81 |
|  |  |  | H | Tyr60 | Gly2 | -3.70 | 2.80 |
|  |  |  | H | Asn62 | Thr8 | -0.80 | 2.94 |
|  |  |  | H | Asn69 | Phe1 | -10.40 | 2.84 |
|  |  |  | H | Gln70 | Ser5 | -2.30 | 2.80 |
|  |  |  | H | Arg71 | Ile6 | -6.20 | 2.77 |
|  |  |  | H | Thr77 | Gly12 | -3.60 | 2.90 |
|  |  |  | H | His81 | Glu15 | -8.30 | 2.71 |
|  |  |  | H | Asn82 | Arg10 | -11.70 | 2.84 |
| VEVRYIDITNILGGV | 5NI9 | –11.92 | I | His13 | Asp7 | -4.28 | 3.66 |
|  |  |  | H | Ser53 | Val15 | -3.90 | 2.69 |
|  |  |  | H | Glu55 | Ile11 | -3.60 | 2.87 |
|  |  |  | H | Glu55 | Gly14 | -5.30 | 2.80 |
|  |  |  | H | Glu55 | Val15 | -0.60 | 3.37 |
|  |  |  | A | Trp61 | Tyr5 | -1.20 | 4.27 |
|  |  |  | H | Asn62 | Asp7 | -5.10 | 2.79 |
|  |  |  | H | Gln64 | Val1 | -7.30 | 2.80 |
|  |  |  | H | Gln64 | Glu2 | -0.50 | 3.60 |
|  |  |  | H | Lys65 | Glu2 | -5.60 | 3.12 |
|  |  |  | H | Asp66 | Glu2 | -8.60 | 2.86 |
|  |  |  | IH | Asp66 | Arg4 | -16.00 | 3.07 |
|  |  |  | H | Gln70 | Asp7 | -4.00 | 2.83 |
|  |  |  | IH | Lys71 | Asp7 | -15.95 | 2.73 |
|  |  |  | H | Lys71 | Ile8 | -3.40 | 3.34 |
|  |  |  | H | Thr77 | Thr9 | -1.40 | 2.95 |
|  |  |  | H | His81 | Asn10 | -1.30 | 2.91 |
|  |  |  | H | Asn82 | Thr9 | -2.30 | 3.04 |
|  |  |  | H | Asn82 | Asn10 | -3.30 | 2.86 |
| MNFIPIIYSKAGKIL | 1AQD | –12.35 | IH | Glu28 | Lys10 | -17.82 | 2.87 |
|  |  |  | H | Tyr60 | Lys13 | -2.80 | 2.72 |
|  |  |  | A | Trp61 | Lys10 | -1.60 | 3.53 |
|  |  |  | H | Asn62 | Ile7 | -1.20 | 3.15 |
|  |  |  | H | Asn62 | Tyr8 | -0.70 | 3.26 |
|  |  |  | H | Ala68 | Lys13 | -7.90 | 2.75 |
|  |  |  | H | Arg71 | Ser9 | -3.70 | 2.85 |
|  |  |  | IH | Glu71 | Lys13 | -17.85 | 2.87 |
|  |  |  | IH | Arg76 | Leu15 | -28.51 | 2.96 |
| SPINIETKKAISDTR | 6CPN | –11.81 | H | Gln9 | Glu6 | -6.10 | 2.82 |
|  |  |  | H | Phe54 | Ser1 | -7.80 | 2.86 |
|  |  |  | IH | Glu55 | Ser1 | -24.22 | 2.69 |
|  |  |  | IH | Asp57 | Arg15 | -28.25 | 2.75 |
|  |  |  | H | Gln57 | Lys9 | -11.30 | 2.78 |
|  |  |  | H | Tyr60 | Thr14 | -3.30 | 2.77 |
|  |  |  | H | Trp61 | Arg15 | -8.50 | 2.88 |
|  |  |  | H | Asn69 | Asp13 | -4.10 | 2.94 |
|  |  |  | H | Asn69 | Thr14 | -0.80 | 3.33 |
|  |  |  | H | Asn69 | Arg15 | -2.50 | 3.07 |
|  |  |  | IH | Asp70 | Lys8 | -26.16 | 2.73 |
|  |  |  | IH | Arg71 | Asp13 | -28.56 | 3.00 |
|  |  |  | IH | Arg76 | Arg15 | -20.26 | 3.04 |
|  |  |  | H | His81 | Asn4 | -2.40 | 2.85 |
| IRDQYITALNHLVLS | 5NI9 | –12.85 | H | Gln9 | Gln4 | -8.50 | 2.82 |
|  |  |  | A | Glu55 | Tyr5 | -0.50 | 4.26 |
|  |  |  | H | Trp61 | Val13 | -2.10 | 3.26 |
|  |  |  | H | Asn62 | Gln4 | -3.30 | 2.82 |
|  |  |  | H | Asn62 | Asn10 | -5.30 | 2.76 |
|  |  |  | H | Gln70 | Ile1 | -8.50 | 2.83 |
|  |  |  | IH | Lys71 | Ser15 | -29.67 | 2.69 |
|  |  |  | H | Thr77 | Ile1 | -6.30 | 2.90 |
|  |  |  | H | Thr77 | Arg2 | -4.00 | 2.87 |
|  |  |  | H | Thr77 | Asp3 | -2.80 | 2.97 |
|  |  |  | H | Asn82 | Asp3 | -4.40 | 2.84 |
| SLPYKYLQVVKQRER | 1AQD | –12.41 | IH | Glu28 | Ser1 | -30.00 | 2.80 |
|  |  |  | H | Arg50 | Arg13 | -6.40 | 2.79 |
|  |  |  | H | Ser53 | Lys11 | -6.30 | 2.85 |
|  |  |  | H | Ser53 | Glu14 | -2.50 | 2.55 |
|  |  |  | H | Gly58 | Tyr4 | -4.20 | 2.71 |
|  |  |  | H | Gln70 | Gln8 | -2.90 | 2.79 |
|  |  |  | H | Arg71 | Pro3 | -2.70 | 2.90 |
|  |  |  | H | Thr77 | Gln8 | -2.00 | 2.69 |
|  |  |  | H | Tyr78 | Leu7 | -0.60 | 3.37 |
|  |  |  | A | His81 | Val10 | -0.70 | 3.54 |
|  |  |  | H | His81 | Lys11 | -4.10 | 2.84 |
|  |  |  | H | Asn82 | Leu7 | -2.30 | 2.98 |
|  |  |  | IH | Glu87 | Arg15 | -32.22 | 2.77 |
|  |  |  | H | Ser88 | Arg15 | -7.90 | 2.78 |

**Table S5.** Atom-atom interactions across TLR4–vaccine construct interface obtained using PDBsum server

| **Bond** | **TLR4-Atom No** | **TLR4-Atom** | **TLR4 Residues** | **Vaccine Atom No** | **Vaccine Atom** | **Vaccine Residues** | **Distance (Å)** |
| --- | --- | --- | --- | --- | --- | --- | --- |
| H-bond | 4749 | NZ | LYS324 | 13774 | O | TYR293 | 2.78 |
| H-bond | 4770 | OD2 | ASP325 | 13790 | NZ | LYS294 | 2.71 |
| H-bond | 4800 | OG | SER327 | 13557 | OH | TYR278 | 2.81 |
| H-bond | 4817 | OH | TYR328 | 13960 | OH | TYR305 | 2.98 |
| H-bond | 5161 | NZ | LYS349 | 10679 | O | ALA83 | 2.77 |
| H-bond | 5202 | NZ | LYS351 | 10628 | O | ALA79 | 2.82 |
| H-bond | 5202 | NZ | LYS351 | 10653 | OE1 | GLU81 | 2.7 |
| Salt-bridge | 4013 | OE2 | GLU278 | 14151 | NZ | LYS315 | 2.73 |
| Salt-bridge | 4770 | OD2 | ASP325 | 13790 | NZ | LYS294 | 2.71 |
| Salt-bridge | 5202 | NZ | LYS 351 | 10653 | OE1 | GLU81 | 2.7 |
| **TLR4 Non-Contact Residues:** GLU278, CYS281, ASN282, ASP299, ILE301, ASP302, ASN305, LYS324, ASP325, SER327, TYR328, ASN329, LYS349, LYS351, ASP371 | | | | **Vaccine Non-Contact Residues:** LYS315, TYR311, VAL312, ILE316, ILE301, TYR305, ALA304, VAL308, TYR293, LYS294, VAL302, ILE298, ALA79, TYR278, PRO77, ALA83, GLU81 | | | |

**Table S6**. Hydrogen bonds at the TLR4–vaccine construct interface (with occupancy ≥ 2%)

| TLR4 residue-atom (1-1494) | Vaccine residue-atom (1495-2077) | No. of frames | Frequency/  Occupancy % | Avg. distance | St.dev |
| --- | --- | --- | --- | --- | --- |
| Glu252-OE2 | Phe314-NZ | 8524 | 77.5 | 2.81 | 0.093 |
| Leu325-NZ | Pro78-O | 1188 | 12.6 | 2.80 | 0.0886 |
| Asp299-OD1 | Ile293-NZ | 1053 | 11.2 | 2.81 | 0.0985 |
| Lys325-NZ | Pro80-OE1 | 478 | 5.09 | 2.79 | 0.0945 |
| Tyr302-OH | Ala304-OH | 407 | 4.33 | 2.83 | 0.102 |
| Arg901-NH2 | Asn285-O | 318 | 3.38 | 2.87 | 0.0884 |

**Table S7.** Complementary DNA sequence of the constructed monkeypox vaccines subjected to in-silico cloning and RNA structure prediction in this study. The N-terminal contains inserted XhoI restriction site, and the C-terminal contains inserted NheI restriction site (shown in bold letters).

| Construct | Nucleotide Sequence |
| --- | --- |
| MPXV  (1755bp) | **CTCGAG**ACCGGTGCTCTGCTGGCTGCTGGTGCTGCTGCTGGTGGTGGTTCTATGAAAAACGCTCGTACCACCCTGATCGCTGCTCCGTCTCCGGCTCCGTGGTAACCGCCGGCTCCGCCGGCTTCTCCGACCCCGACCACCCCGGCTTGGACCCCGACCCCGCCGCCGGCTCCGACCCCGTGGGCTTCTACCCCGACCTGCCCGCCGCCGCCGACCCCGCCGCCGTGGACCCCGCCGCGTCCGCGTGGTCGTCGTCTGCGTCCGCCGACCTGCCCGCCGCCGGGTCCGCGTCTGCCGGAACCGCCGCGTCGTGGTGGTCCGCCGGTTCCGGTTGCTTACTCTGTTAACTGGGACGCTATCGCTCAGTGCGAATCTGGTGCTACCGGTGCTTCTACCCCGGCTACCGCTACCACCGCTGCTTAAGGTTCTCCGCCGGCTCCGGGTGGTCCGACCGCTGCTGCTGCTGCTCGTCAGCGTCAGCAGGGTGGTGCTGACCAGGGTGGTCGTGAACGTGCTGAAGAACCGGGTCACCAGGGTCTGGCTCGTGTTCGTCAGGAAGGTCGTGGTCGTCGTCAGGGTCAGGTTCGTGGTCGTCTGGACCCGTAACGTCCGCCGCGTCGTCGTCAGCCGGAACACGACCACCACGAACGTCTGCGTCGTCTGCTGCACCAGCAGCACCACCGTCCGGAAGGTCGTCTGCACGAACACCACGCTCGTGCTGACCTGCGTCGTCTGCACCGTCTGCAGGAACGTCACCAGCTGCGTCGTCTGCAGGACGAAGGTCACCCGGCTCGTCAGGGTCGTCTGCTGCGTGCTGAACACCACCCGCACCTGCGTCGTCTGCAGGAACGTCAGCACGAACGTCCGCTGCGTCGTCTGCAGGACCTGCGTCACCACCGTCACCGTGGTGCTTACCTGATCGTTATCATCTACGTTTTCAAAAAAATCAAAATGAACTCTGGTCCAGGCCCGGGCTTCGGGGTATACAGCATCCTGACCTCTCGTGGTGGTATCACCGAAGGTCCGGGTCCGGGTGTTGAAGTTCGTTACATCGACATCACCAACATCCTGGGTGGTGTTGGTCCGGGTCCGGGTATGAACTTCATCCCGATCATCTACTCTAAAGCTGGTAAAATCCTGGGTCCGGGTCCGGGTTCTCCGATCAACATCGAAACCAAAAAAGCTATCTCTGAACACCAGGGTCCGCGTCCGCGTCACCAGGGTCCGGTTCACCACCGTCCGGAACCGCCGGGTGCTGAACGTCCGCGTCCGCGTCAGCCGGCTCTGCAGGTTCCGGCTGGTGGTGAAGCTGAAGGTGAAGGTCCGCGTCCGCGTCAGCACCCGCAGGAAGGTGGTCAGCAGCACCACCCGGTTCGTCCGCAGGGTGAACTGCAGCAGGAAGTTCGTGCTCACCGTCCGCCGGACCAGGGTCAGGGTGGTCAGGGTGAAGAACGTTACCCGAAAAAAGTTAACACCCGTCCGCGTCGTCCGGAACGTCCGGCTGAAGCTGAACTGCAGCGTCACCGTCAGGAAGGTGCTCTGGAACAGGAATCTACCGCTGCTACCCGTCGTCCGCGTTCTACCACCACCGCTCGTTCTACCTCTATGAAATGGCTGTCTGACCTGCGTGAAGCTTGCTTCTCTTACTACCAGAAATACAAAAAAGTTTACTGGTACCTGGGTGTTAACAACCTGCCGTACACCCTGGAAGAACTGCTGCCGCAGCAGGAAGCTCCGCCGCCGCCGCCG**GCTAGC** |


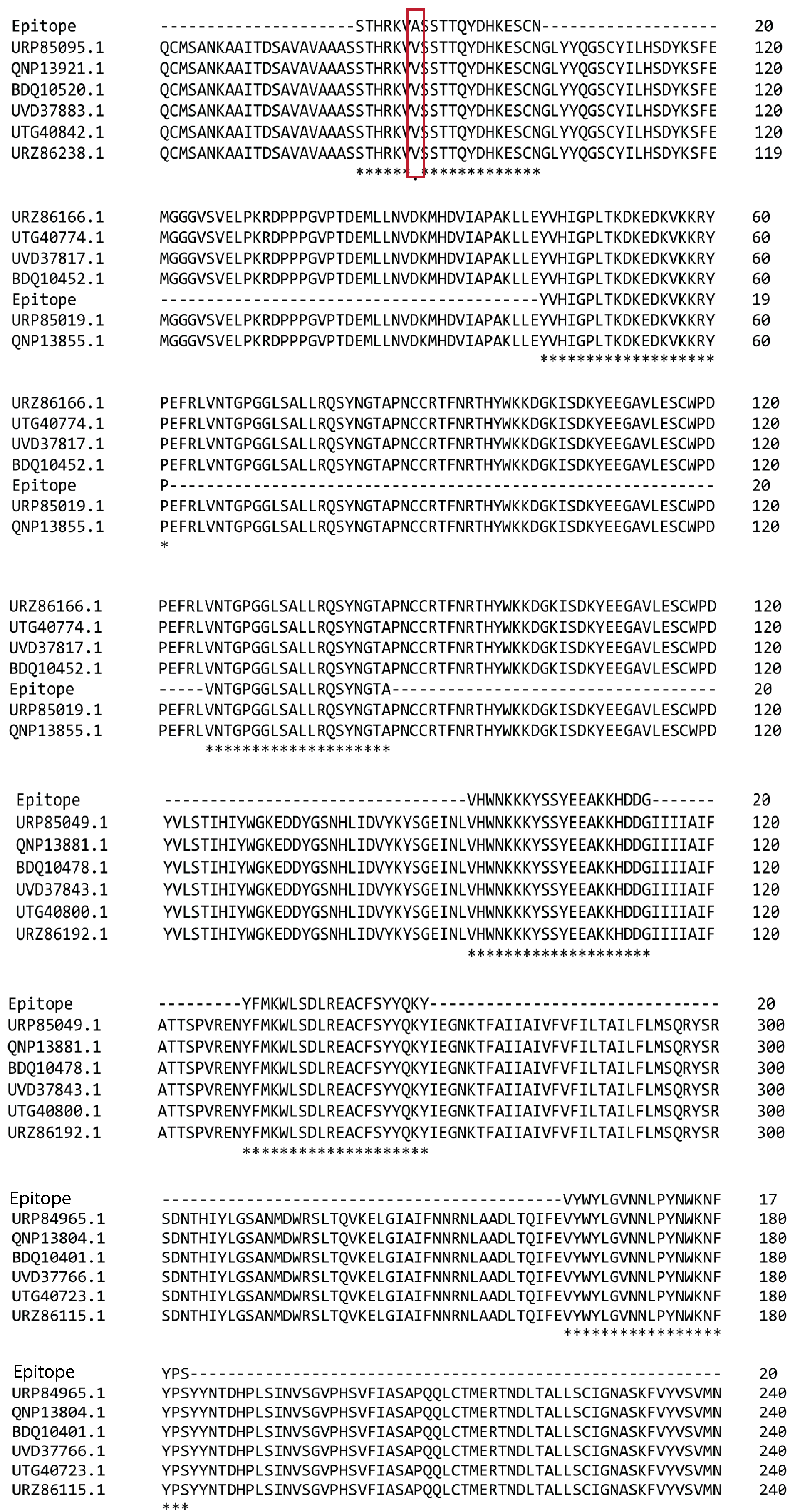


**Figure S1**. Multiple sequence alignment of candidates B-cell epitopes with selected monkeypox isolates using the Clustal Omega program. Only STHRKVASSTTQYDHKESCN of the reference genome is mutated to STHRKVVSSTTQYDHKESCN in all selected MPXV isolates.


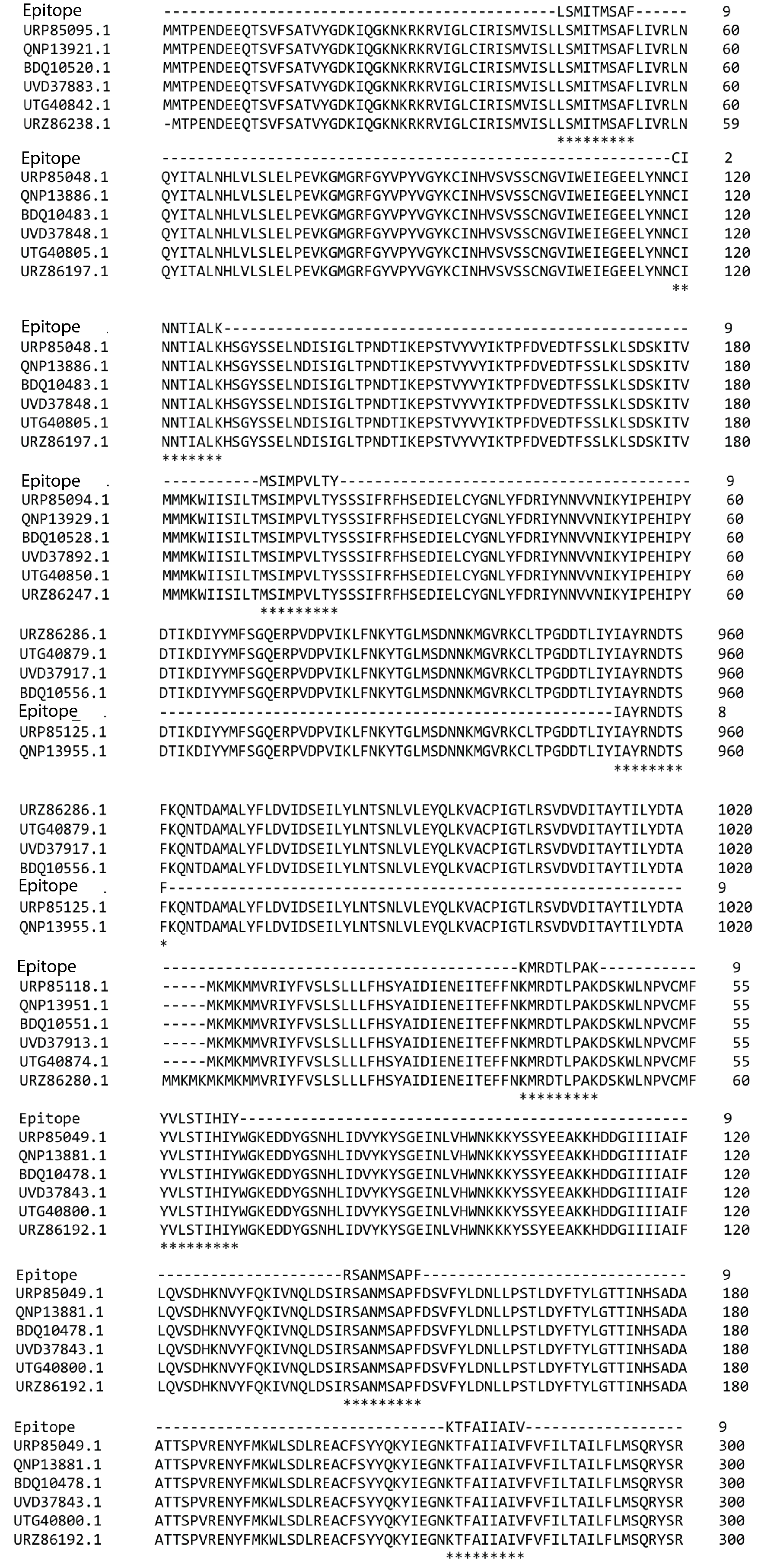


**Figure S2.** Multiple sequence alignment of candidates CTL epitopes with selected monkeypox isolates using the Clustal Omega program. All epitopes perfectly aligned with the same protein sequence from monkeypox isolates showing 100% sequence conservation.


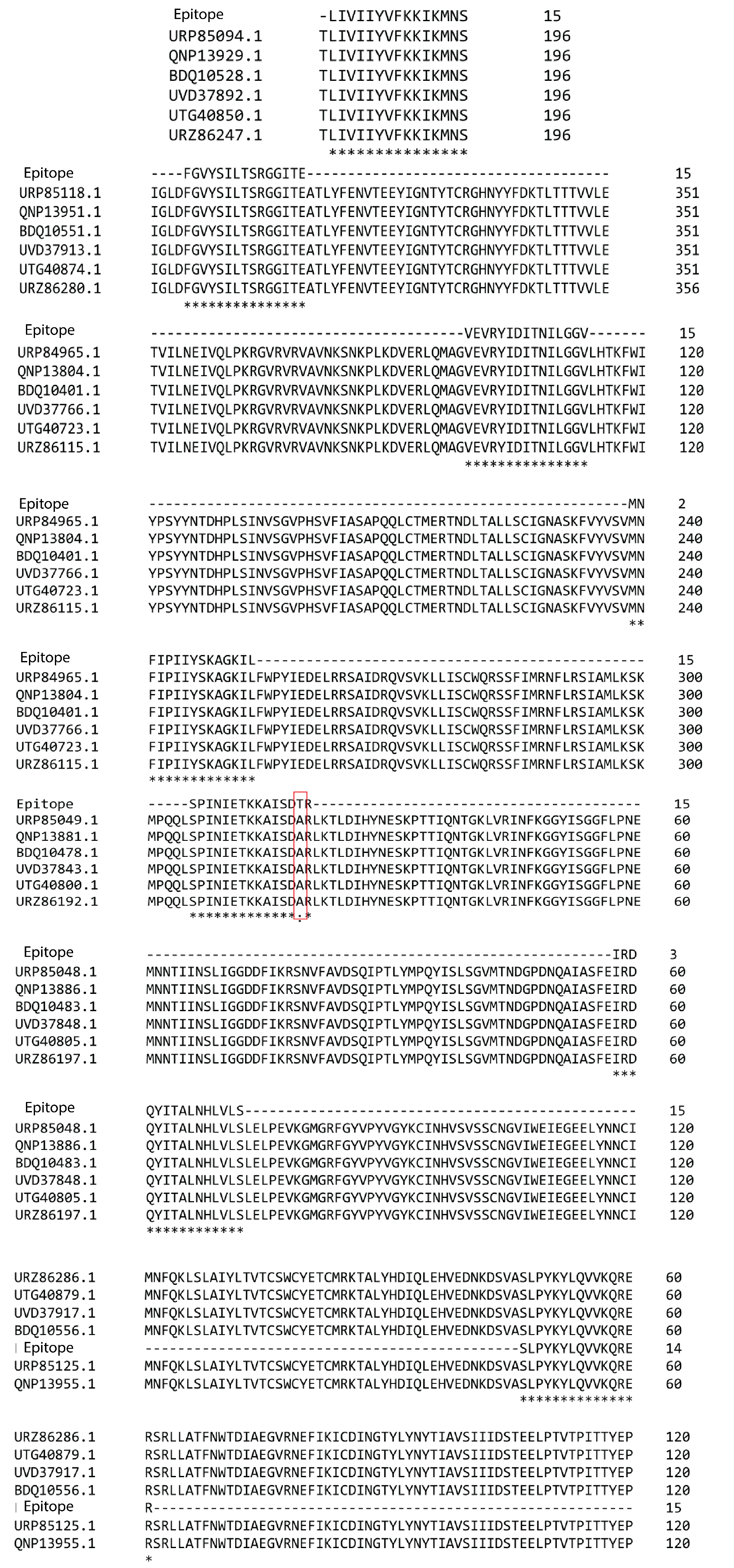


**Figure S3.** Multiple sequence alignment of candidates HTL epitopes with selected monkeypox isolates using the Clustal Omega program. Only SPINIETKKAISD**T**R is mutated to SPINIETKKAISD**A**R in selected monkeypox isolates.

perfectly aligned with the same protein sequence from monkeypox isolates showing 100% sequence conservation.


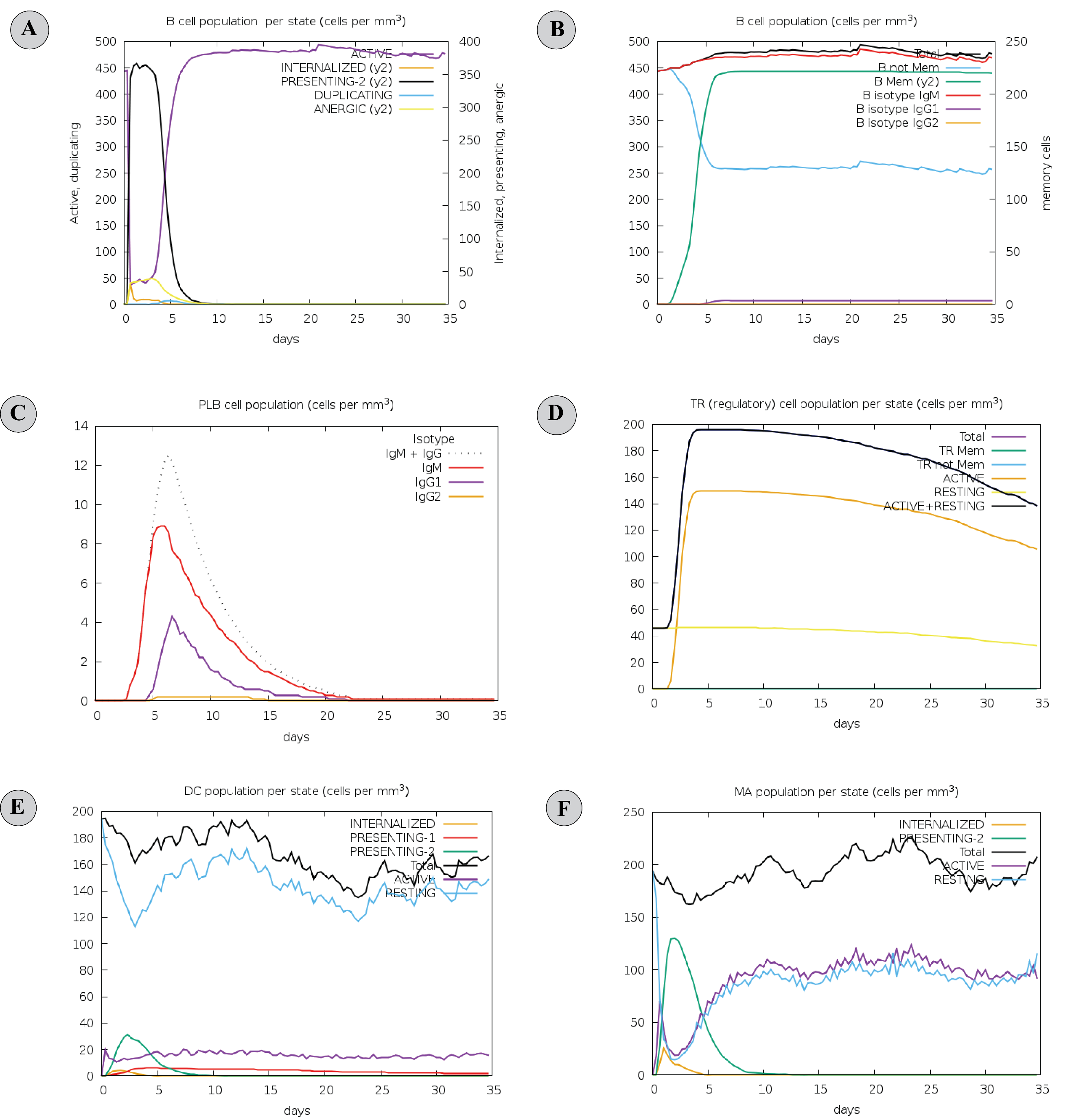


**Figure S4.** In silico immune simulation of an infection challenge, comprised of a virus responding to the sequence of MPXV proteins covered by the multi-epitope construct, was simulated on day 35. **(A)** B lymphocytes population per entity-state (i.e., showing counts for active, presenting on class II, internalized the Ag, duplicating and anergic **(B)** B lymphocytes: total count, memory cells, and sub-divided in isotypes IgM, IgG1 and IgG2 **(C)** Plasma B lymphocytes count sub-divided per isotype (IgM, IgG1 and IgG2) **(D)** CD4+ T-regulatory lymphocytes count. Both total, memory and per entity-state counts are plotted here **(E)** Dendritic cells can present antigenic peptides on both MHC class-I and class-II molecules. The curves show the total number broken down to active, resting, internalized, and presenting the ag **(F)** Total count, internalized, presenting on MHC class-II, active and resting macrophages.


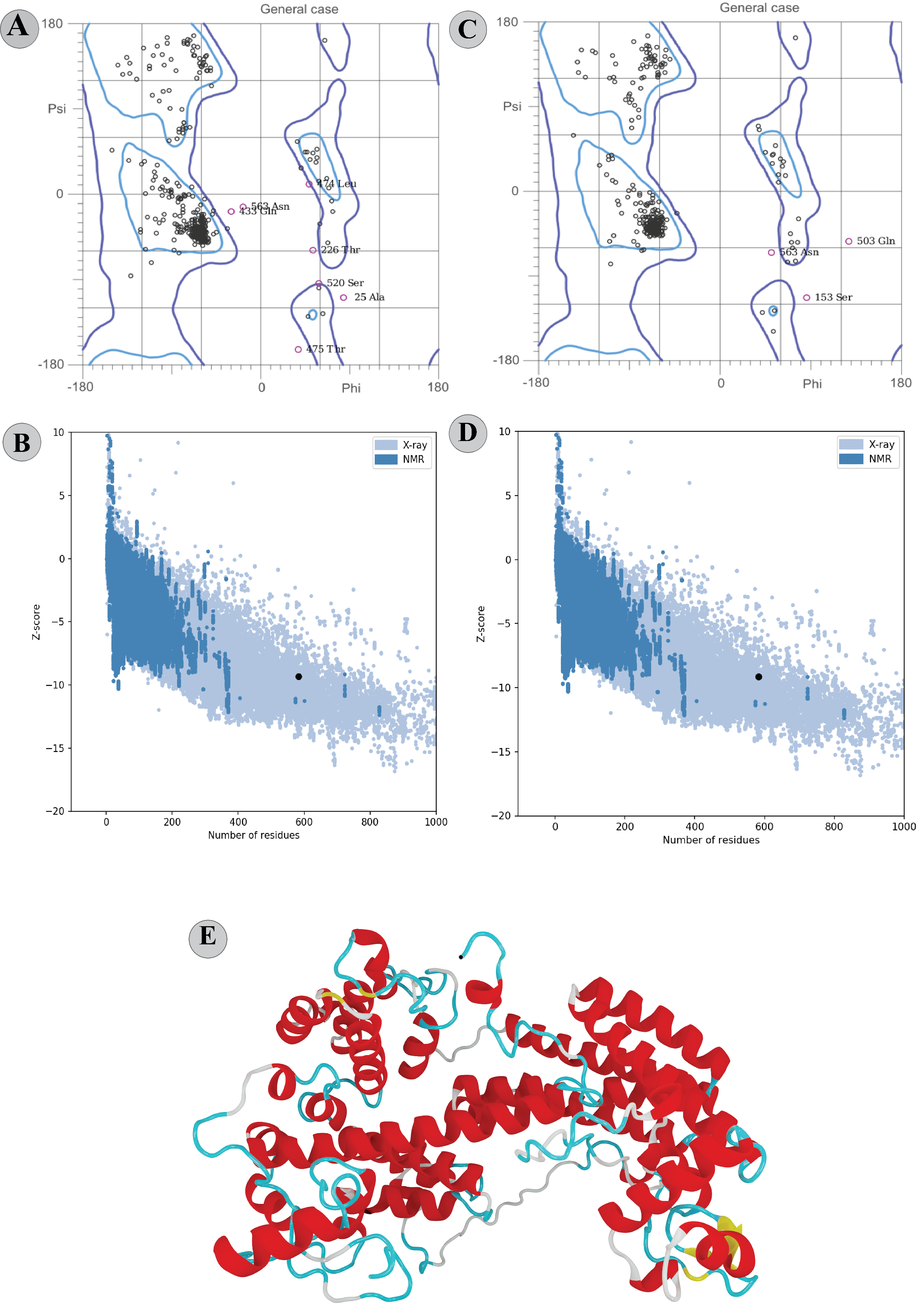


**Figure S5.** Structure validation of the chimeric vaccine’s modeled tertiary structure. Primary modeled structure (before refinement) (A) Ramachandran plot details and **(B**) Z-score graph. Final modeled structure (after refinement), **(C)** Ramachandran plot details, and **(D)** Z-score graph. The Pro-SA web-generated Z-score graph shows the modeled 3D structure corresponding to the X-Ray crystallographic determined structure for the protein of similar sizes. **(E)** 3D structural illustration of the refined modeled vaccine construct.


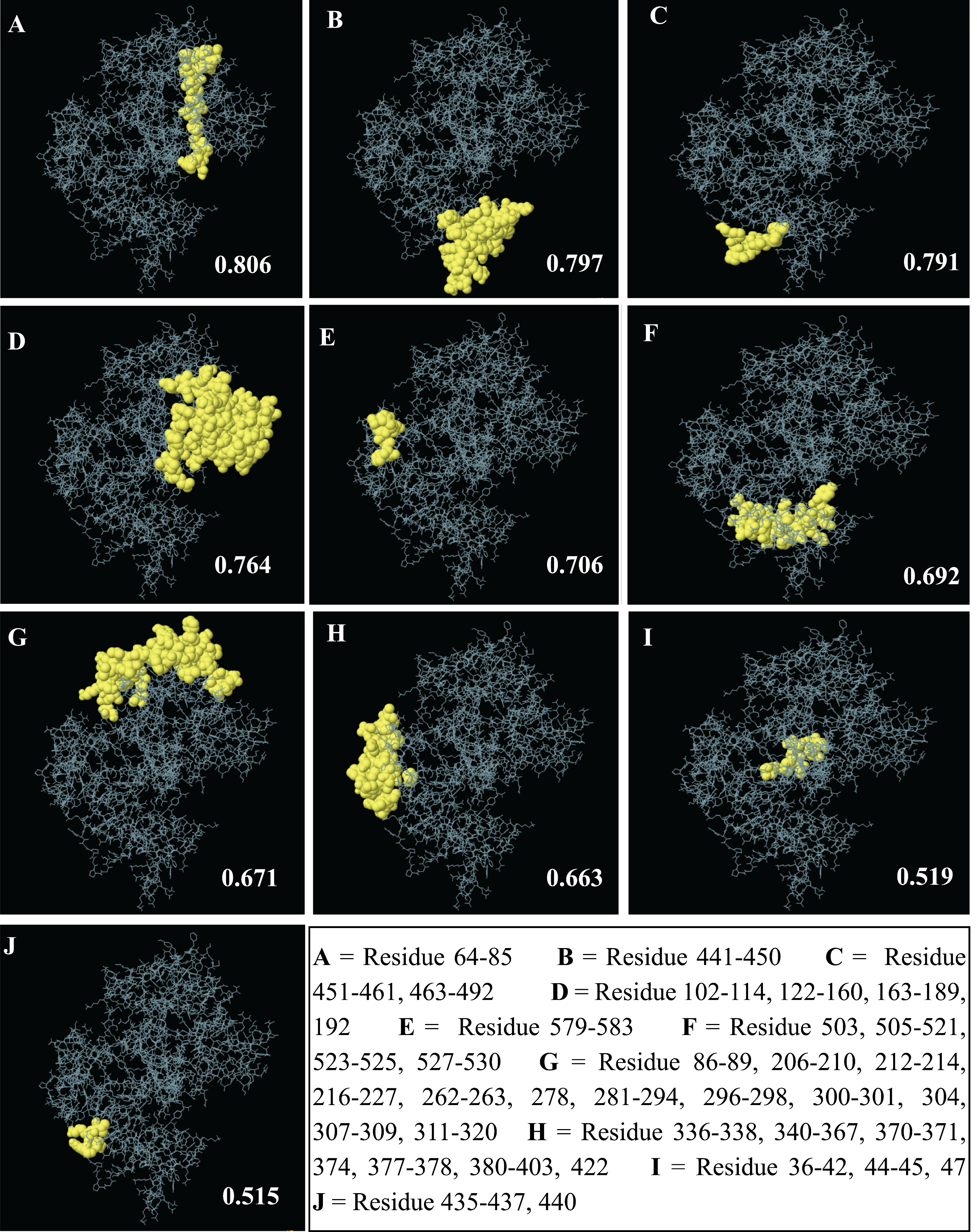


**Figure S6**. Confirmational B-cell epitopes are predicted within the designed vaccine (ElliPro server). The predicted server score is shown with each figure.


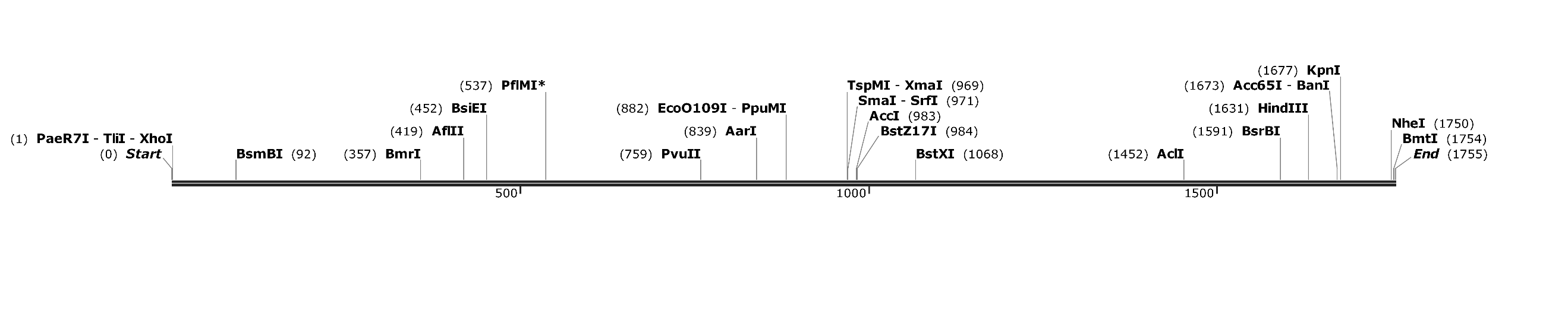
 **Figure S7.** Adapted cDNA construct of the designed vaccine (with restriction enzymes sites added) used for in silico cloning in the expression vector.


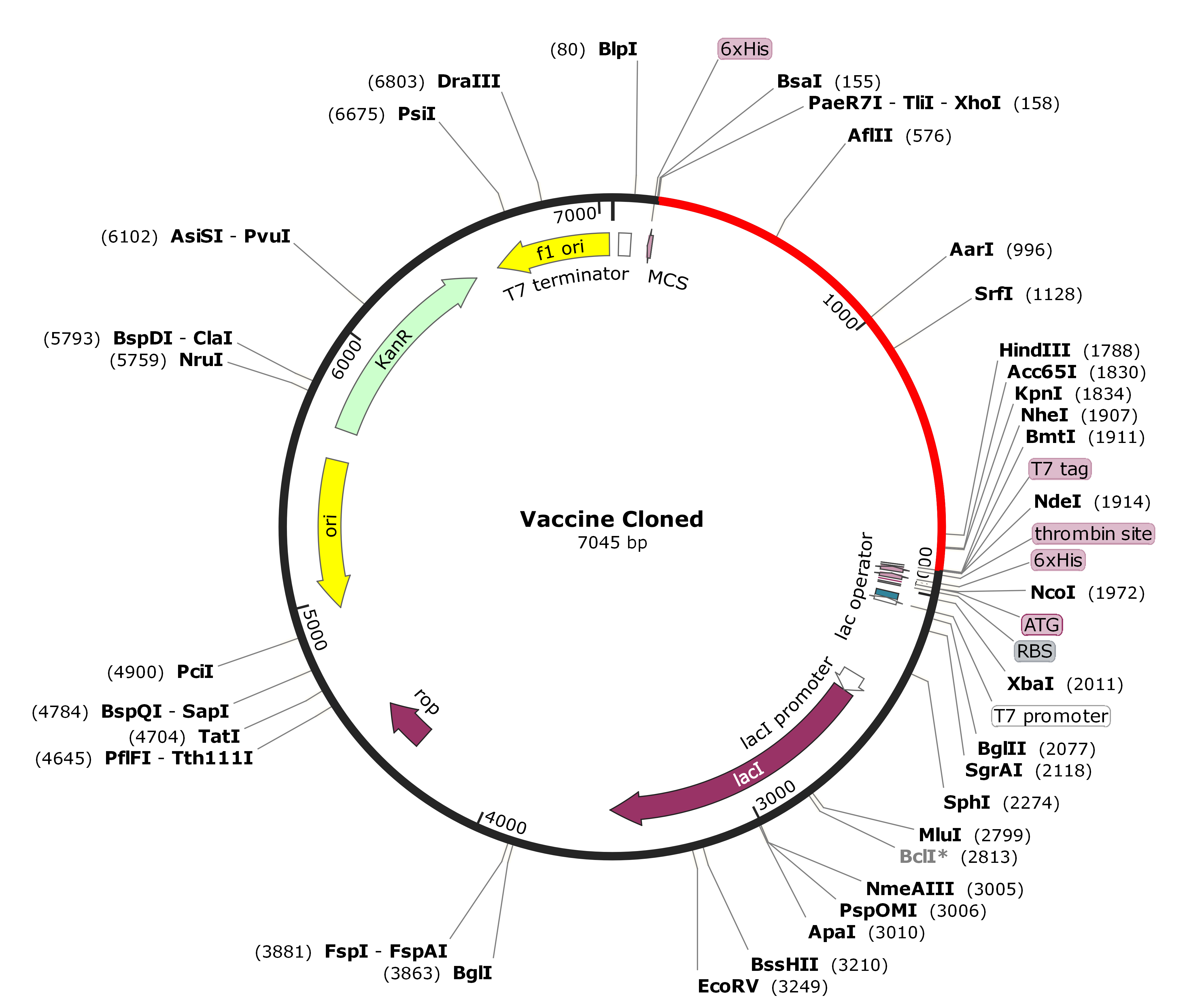


**Figure S8**. Simulated cloning of the designed multi-epitope vaccine construct for MPXV. The adapted DNA sequence of the proposed construct (shown in red) was inserted into the pET-28a (+) expression vector between the XhoI and NheI enzyme loci.


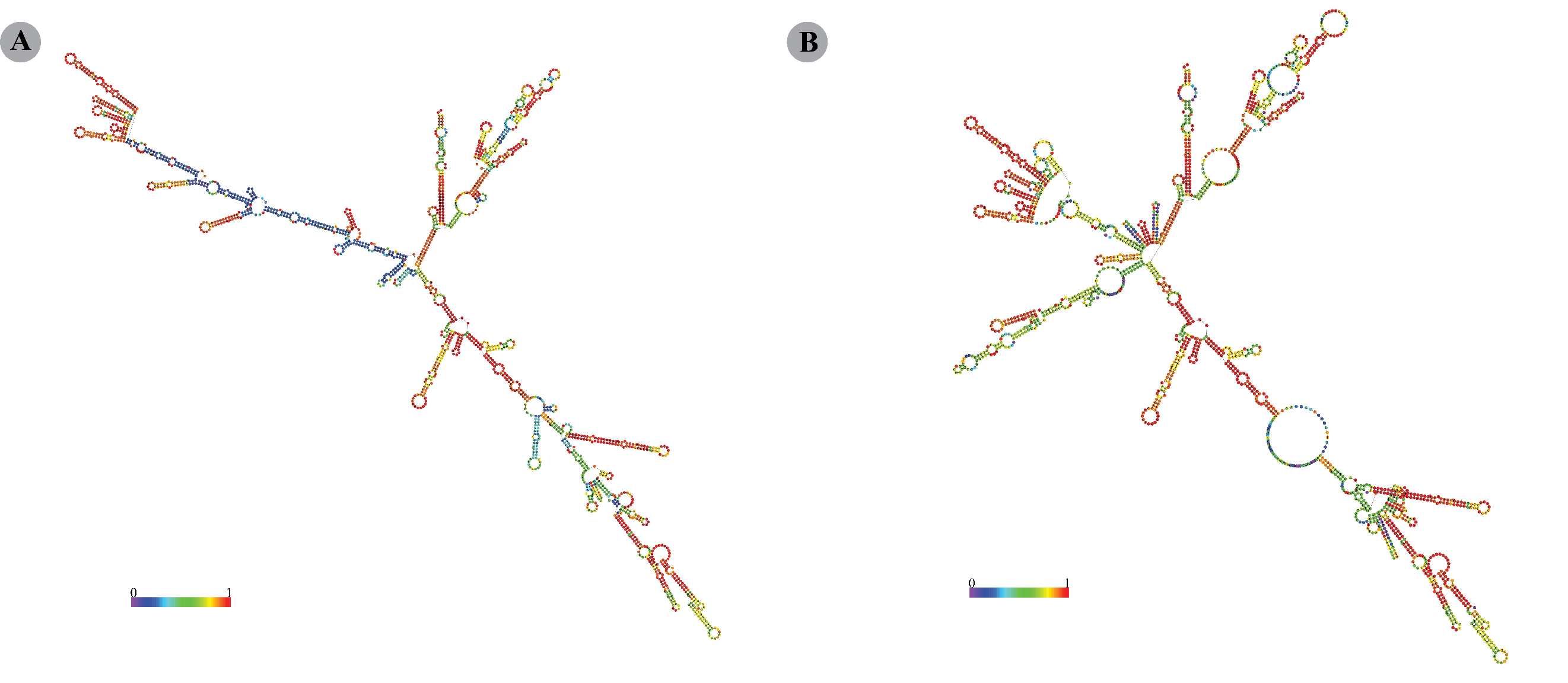


**Figure S9**. Predicted minimum free energy (MFE) mRNA secondary structure (A) and centroid mRNA secondary structure (B) of the designed vaccine (RNAfold program). The MFE structures are colored by base-pairing probabilities. For unpaired regions the color denotes the probability of being unpaired.
